# Generalized Empirical Bayes Modeling via Frequentist Goodness of Fit

**DOI:** 10.1038/s41598-018-28130-5

**Published:** 2018-07-02

**Authors:** Subhadeep Mukhopadhyay, Douglas Fletcher

**Affiliations:** Temple University, Department of Statistical Science, Philadelphia, Pennsylvania 19122 USA

## Abstract

The two key issues of modern Bayesian statistics are: (i) establishing principled approach for *distilling* statistical prior that is *consistent* with the given data from an initial believable scientific prior; and (ii) development of a *consolidated* Bayes-frequentist data analysis workflow that is more effective than either of the two separately. In this paper, we propose the idea of “Bayes *via* goodness-of-fit” as a framework for exploring these fundamental questions, in a way that is general enough to embrace almost all of the familiar probability models. Several examples, spanning application areas such as clinical trials, metrology, insurance, medicine, and ecology show the unique benefit of this new point of view as a practical data science tool.

## Introduction

Bayesians and frequentists have long been ambivalent toward each other^1–3^. The concept of “prior” remains the center of this 250 years old tug-of-war: frequentists view prior as a *weakness* that can hamper scientific objectivity and can corrupt the final statistical inference, whereas Bayesians view it as a *strength* to incorporate relevant domain-knowledge into the data analysis. The question naturally arises: how can we develop a consolidated Bayes-frequentist data analysis workflow^4–7^ that enjoys the best of both worlds? The objective of this paper is to develop one such modeling framework.

We observe samples *y* = (*y*_1_, …, *y*_*k*_) from a known probability distribution *f*(*y*|*θ*), where the unobserved parameters *θ* = (*θ*_1_, …, *θ*_*k*_) are independent realizations from unknown *π*(*θ*). Given such a model, Bayesian inference typically aims at answering the following two questions:

- MacroInference: How should we combine *k* model parameters to come up with an overall, macro-level aggregated statistical behavior of *θ*_1_, …, *θ*_*k*_?
- MicroInference: Given the observables *y*_*i*_, how should we simultaneously estimate individual micro-level parameters *θ*_*i*_?

Thanks to Bayes’ rule, answers to these questions are fairly straightforward and automatic once we have the observed data ${\{{y}_{i}\}}_{i=1}^{k}$ and a specific choice for *π*(*θ*). A common practice is to choose *π* as the parametric conjugate prior *g*(*θ*; *α*, *β*), where the hyper-parameters are either selected based on an investigator’s expert input or estimated from the data (current/historical) when little prior information is available.

## Motivating Questions

However, an applied Bayesian statistician may find it unsatisfactory to work with an initial believable prior *g*(*θ*) at its face value, without being able to interrogate its credibility in the light of the observed data^8,9^ as this choice unavoidably shapes his or her final inferences and decisions. A good statistical practice thus demands greater transparency to address this trust-deficit. What is needed is a justifiable class of prior distributions to answer the following *pre*-inferential modeling questions: Why should I believe your prior? How to check its appropriateness (self-diagnosis)? How to quantify and characterize the uncertainty of the a priori selected *g*? Can we use that information to “refine” the starting prior (*auto*-correction), which is to be used for subsequent inference? In the end, the question remains: how can we develop a systematic and principled approach to go from a *scientific* prior to a *statistical* prior that is consistent with the current data? A resolution of these questions is necessary to develop a “dependable and defensible” Bayesian data analysis workflow, which is the goal of the “Bayes *via* goodness-of-fit” technology.

## Summary of Contributions

This paper provides some practical strategies for addressing these questions by introducing a general modeling framework, along with concrete guidelines for applied users. The major practical advantages of our proposal are: (i) computational ease (it does not require Markov chain Monte Carlo (MCMC), variational methods, or any other sophisticated computational techniques); (ii) simplicity and interpretability of the underlying theoretical framework which is general enough to include *almost all* commonly encountered models; and (iii) easy integration with mainframe Bayesian analysis that makes it readily applicable to a wide range of problems. The next section introduces a new class of nonparametric priors DS(*G*, *m*) along with its role in exploratory graphical diagnostic and uncertainty quantification. The estimation theory, algorithm, and real data examples are discussed in Section 2. Consequences for inference are discussed in Section 3, which include methods of combining heterogeneous studies and a generalized nonparametric Stein-prediction formula that selectively borrows strength from ‘similar’ experiments in an automated manner. Section 3.2 describes a new theory of ‘learning from uncertain data,’ which is an important problem in many application fields including metrology, physics, and chemistry. Section 3.4 solves a long-standing puzzle of modern empirical Bayes, originally posed by Herbert Robbins^10^. We conclude the paper with some final remarks in Section 4. Connections with other Bayesian cultures are presented in the supplementary material to ensure the smooth flow of main ideas.

## Real-Data Applications

To demonstrate the versatility of the proposed “Bayes *via* goodness-of-fit” data analysis scheme, we selected examples from a wide range of models including normal, Poisson, and Binomial distributions. The full catalog of datasets is presented in Supplementary Table 2.

## Notation

The notation *g* and *G* denote the density and distribution function of the starting prior, while *π* and Π denote the density and distribution function of the unknown oracle prior. We will denote the conjugate prior with hyperparameters *α* and *β* by *g*(*θ*; *α*, *β*). Let ${ {\mathcal L} }^{2}(\mu )$ be the space of square integrable functions with inner product $\int \,f(u)g(u)\,{\rm{d}}\mu (u)$. Leg_*j*_(*u*) denotes *j*th shifted orthonormal Legendre polynomials on $[0,1]$. They form a complete orthonormal basis for ${ {\mathcal L} }^{2}(0,1)$. Whereas ${T}_{j}(\theta ;G):\,={{\rm{Leg}}}_{j}[G(\theta )]$ is the modified shifted Legendre polynomials of rank-G transform *G*(*θ*), which are basis of the Hilbert space ${ {\mathcal L} }^{2}(G)$. The composition of functions is denoted by the usual ‘$\circ$’ sign.

## The Model

Our model-building approach proceeds sequentially as follows: (i) it starts with a scientific (or empirical) parametric prior *g*(*θ*; *α*, *β*), (ii) inspects the adequacy and the remaining uncertainty of the elicited prior using a graphical exploratory tool, (iii) estimates the necessary “correction” for assumed *g* by looking at the data, (iv) generates the final statistical estimate $\hat{\pi }(\theta )$, and (v) executes macro and micro-level inference. We seek a method that can yield answers to all five of the phases using only a *single* algorithm.

### New Family of Prior Densities

This section serves two purposes: it provides a universal class of prior density models, followed by its Fourier non-parametric representation in a specialized orthonormal basis.

#### **Definition 1**

. The Skew-G class of density models is given by

1.1 $$\pi (\theta )=g(\theta ;\alpha ,\beta )\,d[G(\theta );G,{\rm{\Pi }}],$$

where $d(u;G,{\rm{\Pi }})=\pi ({G}^{-1}(u))/g({G}^{-1}(u))$ *for* 0 < *u* < 1 and consequently ${\int }_{0}^{1}\,d(u;G,{\rm{\Pi }})=1$.

A few notes on the model specification:

- It has a unique *two*-*component* structure that combines assumed parametric *g* with the *d*-function. The function *d* can be viewed as a “correction” density to counter the possible misspecification bias of *g*.
- The density function *d*(*u*; *G*, Π) can also be viewed as describing the “excess” *uncertainty* of the assumed *g*(*θ*; *α*, *β*). For that reason we call it the U-function.
- The motivation behind the representation (1.1) stems from the observation that *d*[*G*(*θ*); *G*, Π] is in fact the prior density-ratio *π*(*θ*)/*g*(*θ*). Hence, it is straightforward to verify that the scheme (1.1) always yields a proper density, i.e., ${\int }_{\theta }\,g(\theta )\,d[G(\theta );G,{\rm{\Pi }}]=1$.
  Since the square integrable *d*[*G*(*θ*); *G*, Π] lives in the Hilbert space ${ {\mathcal L} }^{2}(G)$, we can approximate it by projecting into the orthonormal basis {*T*_*j*_} satisfying $\int \,{T}_{i}(\theta ;G){T}_{j}(\theta ;G)\,{\rm{d}}G={\delta }_{ij}$. We choose *T*_*j*_(*θ*; *G*) to be ${{\rm{Leg}}}_{j}\circ G(\theta )$, a member of the LP-class of rank-polynomials^11^. The system {*T*_*j*_} possesses two attractive properties: they are polynomials of rank transform *G*(*θ*) thus constitutes a robust basis, and they are orthonormal with respect to ${ {\mathcal L} }^{2}(G)$, for *any* arbitrary *G* (continuous). This is not to be confused with standard Legendre polynomials Leg_*j*_(*u*), 0 < *u* < 1, which are orthonormal with respect to Uniform$[0,1]$ measure. For more details, see Supplementary Appendix B. The above discussion paves the way for the following definition.
  **Definition 2**
  . Θ ~ DS(*G*, *m*) distribution if it admits the following representation:
  1.2 $$\pi (\theta )\,=\,g(\theta ;\alpha ,\beta )\,[1+\sum _{j=1}^{m}\,{\rm{LP}}[j;G,{\rm{\Pi }}]\,{T}_{j}(\theta ;G)].$$
  The LP-Fourier coefficients LP[*j*; *G*, Π] are the key parameters that help us to express mathematically the “gap” between a priori anticipated *G* and the true prior Π. When all the expansion coefficients are zero, we automatically recover *g*.
- When *π*(*θ*) is a member of DS(*G*, *m*) class of priors, the orthogonal LP-transform coefficients (1.2) satisfy
  1.3 $${\rm{LP}}[j;G,{\rm{\Pi }}]={\langle d,{T}_{j}\circ {G}^{-1}\rangle }_{{ {\mathcal L} }^{2}(0,1)}={\mathbb{E}}[{T}_{j}({\rm{\Theta }};G);{\rm{\Pi }}].$$
  Thus, given a random sample *θ*_1_, …, *θ*_*k*_ from *π*(*θ*), we could easily estimate the unknown LP-coefficients, and, thus, *d* and *π*, by computing the sample mean ${k}^{-1}\,{\sum }_{i=1}^{k}\,{T}_{j}({\theta }_{i};G)$. *But unfortunately*, *the θ*_*i*_’*s are unobserved*. Section 2 describes an estimation strategy that can deal with the situation at hand. Before introducing this technique, however, we must acclimate the reader with the role played by the U-function *d*(*u*; *G*, Π) for uncertainty quantification and characterization of the initial believable prior *g*. That’s the objective of the next Section 1.2.
- Under definition 2, we have DS(*G*, *m* = 0) ≡ *g*(*θ*; *α*, *β*). The truncation point *m* in (1.2) reflects the *concentration* of permissible *π* around a known *g*. While this class of priors is rich enough to approximate any reasonable prior with the desired accuracy in the large-*m* limit, one can easily exclude absurdly rough densities and focus on a neighborhood around the domain-knowledge-based *g* by choosing *m* not “too big.”
- The motivations behind the name ‘DS-Prior’ are twofold. First, our formulation operationalizes I. J. Good’s ‘Successive Deepening’ idea^12^ for Bayesian data analysis:

*A hypothesis is formulated*, *and*, *if it explains enough*, *it is judged to be probably approximately correct*. *The next stage is to try to improve it*. *The form that this approach often takes in EDA is to examine residuals for patterns*, *or to treat them as if they were original data* (I. J. Good, 1983, p. 289).

Secondly, our prior has two components: A Scientific *g* that encodes an expert’s knowledge and a Data-driven *d*. That is to say that our framework embraces data and science, both, in a *testable* manner^13^.

### Exploratory Diagnostics and U-Function

**Figure 1:**
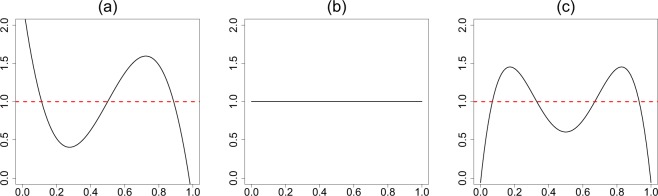
Graphical diagnostic tool: U-functions for (**a**) rat tumor data; (**b**) terbinafine and ulcer data; (**c**) rolling tacks data. The deviation from uniformity (red dotted line) indicates that the default prior contradicts the observed data. The flat shape of the U-function in panel (b) suggests Beta(1.24, 34.7) and ${\mathscr{N}}(\,-\,1.17,\,0.98)$ are consistent with the terbinafine and ulcer data, respectively.

Is your data compatible with the pre-selected *g*(*θ*)? If yes, the job is done without getting into the arduous business of nonparametric estimation. If no, we can model the “gap” between the parametric *g* and the true unknown prior *π*, which is often *far easier* than modeling *π* from scratch (hence, one can learn from small number of cases)! If the observed *y*_1_, …, *y*_*k*_ look very unexpected given *g*(*θ*; *α*, *β*), it is completely reasonable to question the sanctity of such a self-selected prior. Here we provide a formal nonparametric exploratory procedure to describe comprehensively the uncertainty about the choice of *g*. Using the algorithm detailed in the next section, we estimate U-functions for four real data sets. Among them, the first three are binomial variate and the last one normal. The results are shown in Fig. 1.

- The rat tumor data^14^ consists of observations of endometrial stromal polyp incidence in *k* = 70 groups of female rats. For each group, *y*_*i*_ is the number of rats with polyps and *n*_*i*_ is the total number of rats in the experiment.
- The terbinafine data^15^ comprise *k* = 41 studies, which investigate the proportion of patients whose treatment terminated early due to some adverse effect of an oral anti-fungal agent: *y*_*i*_ is the number of terminated treatments and *n*_*i*_ is the total number of patients in the experiment.
- The rolling tacks^16^ data involve flipping a common thumbtack 9 times. It consists of 320 pairs, (9, *y*_*i*_), where *y*_*i*_ represents the number of times the thumbtack landed point up.
- The ulcer data consist of forty randomized trials of a surgical treatment for stomach ulcers conducted between 1980 and 1989^17,18^. Each of the 40 trials has an estimated log-odds ratio ${y}_{i}|{\theta }_{i}\sim {\mathscr{N}}({\theta }_{i},{s}_{i}^{2})$ that measures the rate of occurrence of recurrent bleeding given the surgical treatment.
  Throughout, we have used the maximum likelihood estimates (MLE) for estimating the initial starting value of the hyperparameters. However, one can use any other reasonable choice, which may involve expert’s judgment. What is important to note is the *shape* of the $\hat{d}$; more specifically, its departure from uniformity, indicates the assumed conjugate prior *g*(*θ*; *α*, *β*) needs a ‘repair’ to resolve the prior-data conflict. For example, the flat shape of the estimated $\hat{d}$ in Fig. 1(b) indicates that our initial selection of *g*(*θ*; *α*, *β*) is appropriate for the terbinafine and ulcer data. Therefore, one can proceed in turning the “Bayesian crank” with confidence using the parametric beta and normal prior respectively.
  In contrast, Fig. 1(a,c) provide a strong warning in using *g* = Beta(*α*, *β*) for the rat tumor and the rolling tacks experiments. The smooth estimated U-functions expose the nature of the discrepancy that exists between *g* and the observed data by having an “extra” mode. Clearly, the answer does not lie in choosing a different (*α*, *β*) as this cannot rectify the missing bimodality. This brings us to an important point: the full Bayesian analysis, by assigning hyperprior distribution on *α* and *β*, is not always a fail-safe strategy and should be practiced with caution (not in a blind mechanical way). The bottom line is uncertainty in the prior probability model ≠ uncertainty in *α*, *β*. A foolproof prior uncertainty model, thus, has to allow ignorance in terms of the *functional shape* around *g*. The foregoing discussion motivates the following entropy-like measure of uncertainty.
  **Definition 3**
  . The *q*LP statistic for uncertainty quantification is defined as follows:
  4 $${\rm{qLP}}(G\parallel {\rm{\Pi }})=\sum _{j}\,|{\rm{LP}}[j;G,{\rm{\Pi }}]{|}^{2}.$$
  The motivation behind this definition comes from applying Parseval’s identity in (1.2): $${\int }_{0}^{1}\,{d}^{2}(u;G,{\rm{\Pi }})=$$$$1+{\rm{qLP}}(G\parallel {\rm{\Pi }})$$. Thus, the proposed measure captures the departure of the U-function from uniformity. The following result connects our *q*LP statistic with relative entropy.
  **Theorem 1**
  . *The q*LP *uncertainty quantification statistic satisfies the following relation*:
  5 $${\rm{qLP}}(G\parallel {\rm{\Pi }})\approx 2\times {\rm{KL}}({\rm{\Pi }}\parallel G),$$
  *where* KL(Π||*G*) *is the Kullback*–*Leibler* (*KL*) *divergence between the true prior π and its parametric approximate g*.
  **Proof**
  . Express KL-information divergence using U-functions by substituting *G*(*θ*) = *u*:
  6 $${\rm{KL}}({\rm{\Pi }}\parallel G)=\int \,\pi (\theta )\,\mathrm{log}\,\frac{\pi (\theta )}{g(\theta )}\,{\rm{d}}\theta ={\int }_{0}^{1}\,d(u;G,{\rm{\Pi }})\,\mathrm{log}\,d(u;G,{\rm{\Pi }})\,{\rm{d}}u.$$
  Complete the proof by approximating *dlogd* in (1.6) via Taylor series $(d-1)+\frac{1}{2}{(d-1)}^{2}$.
- Our exploratory uncertainty diagnostic tool encourages “interactive” data analysis that is similar in spirit to Gelman *et al*.^19^. Subject-matter experts can use this tool to “play” with different hyperparameter choices in order to filter out the reasonable ones. This functionality might be especially valuable when multiple expert opinions are available.
- When $\hat{d}$ shows evidence of the prior-data conflict, the question remains: what to do next? It is not enough to check the adequacy without informing the user an explanation for the misfit or what is the “deeper” structure that is missing in the starting parametric prior. Fortunately, our DS(*G*, *m*) model suggests a simple, yet formal, guideline for upgrading: $\widehat{\pi }(\theta )=g(\theta ;\hat{\alpha },\hat{\beta })\times \hat{d}[G(\theta );G,{\rm{\Pi }}]$, where the shape of $\hat{d}(u;G,{\rm{\Pi }})$ captures the patterns which were not a priori anticipated. Hence our formalism *simultaneously* addresses the problem of uncertainty quantification and the subsequent model synthesis.

## Estimation Method

### Theory

In this Section, we lay out the key theoretical results that we use for designing our algorithm. Before deriving the general expressions under the LP-DS(*G*, *m*) model, it is helpful to start by recalling the results for the basic conjugate model, i.e., Θ ~ DS(*G*, *m* = 0) and ${y}_{i}|{\theta }_{i}\mathop{\sim }\limits^{{\rm{ind}}}f({y}_{i}|{\theta }_{i})$ for *i* = 1, …, *k*. Table 1 provides the marginal ${f}_{G}({y}_{i})={\int }_{{\theta }_{i}}\,f({y}_{i}|{\theta }_{i})g({\theta }_{i})\,{\rm{d}}{\theta }_{i}$ and the posterior distribution ${\pi }_{G}({\theta }_{i}|{y}_{i})=\frac{f({y}_{i}|{\theta }_{i})g({\theta }_{i})}{{f}_{G}({y}_{i})}$ for four commonly encountered distributions, with the Bayes estimate of *h*(Θ_*i*_) being denoted as ${{\mathbb{E}}}_{G}[h({{\rm{\Theta }}}_{i})|{y}_{i}]={\int }_{{\theta }_{i}}\,h({\theta }_{i}){\pi }_{G}({\theta }_{i}|{y}_{i})\,{\rm{d}}{\theta }_{i}$. The subscript ‘*G*’ in these expressions underscores the fact that they are calculated for the conjugate *g*-model.

**Table 1:** Details on the distributions, their conjugate priors, and the resulting marginal and posterior distributions for four familiar distributions (two discrete and two continuous): Binomial, Poisson, Normal, and Exponential.

Family	Conjugate *g*-prior	Marginal [*f*_*G*_(*y*_*i*_)]	Posterior [*π*_*G*_(*θ*_*i*_|*y*_*i*_)]
Binomial(*n*_*i*_, *θ*_*i*_)	Beta(*α*, *β*)	$$(\begin{array}{c}{n}_{i}\\ {y}_{i}\end{array})\frac{{\bf{B}}(\alpha +{y}_{i},\beta -{y}_{i}+{n}_{i})}{{\bf{B}}(\alpha ,\beta )}$$	Beta(*α* + *y*_*i*_, *β* − *y*_*i*_ + *n*_*i*_)
Poisson(*θ*_*i*_)	Gamma(*α*, *β*)	$$(\begin{array}{c}{y}_{i}+\alpha -1\\ {y}_{i}\end{array}){p}^{\alpha }{(1-p)}^{{y}_{i}}$$	${\rm{Gamma}}(\alpha +{y}_{i},\frac{\beta }{1+\beta })$
${\rm{Normal}}({\theta }_{i},{\sigma }_{i}^{2})$	Normal(*α*, *β*^2^)	${\rm{Normal}}(\alpha ,{\sigma }_{i}^{2}+{\beta }^{2})$	${\rm{Normal}}({\lambda }_{i}\alpha +(1-{\lambda }_{i}){y}_{i},(1-{\lambda }_{i}){\sigma }_{i}^{2})$
Exp(*λ*)	Gamma(*α*, *β*)	$\frac{\alpha \beta }{{(1+\beta y)}^{\alpha +1}}$	${\rm{Gamma}}(\alpha +1,\frac{\beta }{1+\beta {y}_{i}})$

For the normal-normal posterior ${\lambda }_{i}={\sigma }_{i}^{2}/({\sigma }_{i}^{2}+{\beta }^{2})$ and in the marginal of the Poisson-gamma *p* = 1/(1 + *β*). We use ${\bf{B}}(\alpha ,\beta )=\frac{{\rm{\Gamma }}(\alpha ){\rm{\Gamma }}(\beta )}{{\rm{\Gamma }}(\alpha +\beta )}$ to denote the normalizing constant of beta distribution.

Next, we seek to extend these parametric results to LP-nonparametric setup in a systematic way. Especially, without deriving analytical expressions for each case separately, we want to establish a more general representation theory that is valid for all of the above and, in fact, extends to any conjugate pairs, explicating the underlying unity of our formulation.

#### Theorem 2.

*Consider the following model*:

$$\begin{array}{lll}{y}_{i}|{\theta }_{i} & \mathop{\sim }\limits^{{\rm{ind}}} & f({y}_{i}|{\theta }_{i}),\,(i=1,\ldots ,k)\\ {{\rm{\Theta }}}_{i} & \mathop{\sim }\limits^{{\rm{ind}}} & \pi (\theta ),\end{array}$$

where *π*(*θ*) *is a member of* DS(*G*, *m*) *family* (1.2), *G being the associated conjugate prior*. *Under this framework*, *the following holds*:

- *The marginal distribution of y*_*i*_ *is given by*
  2.1 $${f}_{{\rm{LP}}}({y}_{i})={f}_{G}({y}_{i})\,(1+\sum _{j}\,{\rm{LP}}[j;G,{\rm{\Pi }}]\,{{\mathbb{E}}}_{G}[{T}_{j}({{\rm{\Theta }}}_{i};G)\,|{y}_{i}]),$$
  *where* $${{\mathbb{E}}}_{G}[{T}_{j}({{\rm{\Theta }}}_{i};G)|{y}_{i}]={\int }_{{\theta }_{i}}\,{{\rm{Leg}}}_{j}\circ G({\theta }_{i}){\pi }_{G}({\theta }_{i}|{y}_{i})\,{\rm{d}}{\theta }_{i}$$.
- *A closed*-*form expression for the posterior distribution of* Θ_*i*_ *given y*_*i*_ *is*
  2.2 $${\pi }_{{\rm{LP}}}({\theta }_{i}|{y}_{i})=\frac{{\pi }_{G}({\theta }_{i}|{y}_{i})\,(1+{\sum }_{j}\,{\rm{LP}}[j;G,{\rm{\Pi }}]\,{T}_{j}({\theta }_{i};G))}{1+{\sum }_{j}\,{\rm{LP}}[j;G,{\rm{\Pi }}]\,{{\mathbb{E}}}_{G}[{T}_{j}({{\rm{\Theta }}}_{i};G)|{y}_{i}]}$$
- *For any general random variable h*(Θ_*i*_), *the Bayes conditional mean estimator can be expressed as follows*:

2.3 $${{\mathbb{E}}}_{{\rm{LP}}}[h({{\rm{\Theta }}}_{i})|{y}_{i}]=\frac{{{\mathbb{E}}}_{G}[h({{\rm{\Theta }}}_{i})|{y}_{i}]+{\sum }_{j}\,{\rm{LP}}[j;G,{\rm{\Pi }}]\,{{\mathbb{E}}}_{G}[h({{\rm{\Theta }}}_{i}){T}_{j}({{\rm{\Theta }}}_{i};G)|{y}_{i}]}{1+{\sum }_{j}\,{\rm{LP}}[j;G,{\rm{\Pi }}]\,{{\mathbb{E}}}_{G}[{T}_{j}({{\rm{\Theta }}}_{i};G)|{y}_{i}]}$$

#### *Proof*.

The marginal distribution for DS(*G*, *m*)-nonparametric model can be represented as:

$${f}_{{\rm{LP}}}({y}_{i})=\int \,f({y}_{i}|{\theta }_{i})\times \{g({\theta }_{i};\alpha ,\beta )\,d[G({\theta }_{i});G,{\rm{\Pi }}]\}\,{\rm{d}}{\theta }_{i}.$$

Expanding the U-function in the LP-bases (1.2) yields

2.4 $${f}_{{\rm{LP}}}({y}_{i})={f}_{G}({y}_{i})+\sum _{j}\,{\rm{LP}}[j;G,{\rm{\Pi }}]\,\int \,{T}_{j}({\theta }_{i};G)f({y}_{i}|{\theta }_{i})g({\theta }_{i};\alpha ,\beta )\,{\rm{d}}{\theta }_{i}.$$

The next step is to recognize that

2.5 $$f({y}_{i}|{\theta }_{i})\,g({\theta }_{i};\alpha ,\beta )={f}_{G}({y}_{i})\,{\pi }_{G}({\theta }_{i}|{y}_{i}).$$

Substituting (2.5) in the second term of (2.4) leads to

2.6 $$\sum _{j}\,{\rm{LP}}[j;G,{\rm{\Pi }}]\,\int \,{T}_{j}({\theta }_{i};G)f({y}_{i}|{\theta }_{i})g({\theta }_{i};\alpha ,\beta )\,{\rm{d}}{\theta }_{i}={f}_{G}({y}_{i})\,\sum _{j}\,{\rm{LP}}[j;G,{\rm{\Pi }}]\,{{\mathbb{E}}}_{G}[{T}_{j}({{\rm{\Theta }}}_{i};G)|{y}_{i}].$$

Complete the proof of part (a) by replacing (2.6) into (2.4).

For part (b) of posterior distribution calculation we have

2.7 $${\pi }_{{\rm{LP}}}({\theta }_{i}|{y}_{i})=\frac{f({y}_{i}|{\theta }_{i})\,g({\theta }_{i};\alpha ,\beta )}{{f}_{{\rm{LP}}}({y}_{i})}\{1+\sum _{j}\,{\rm{LP}}[j;G,{\rm{\Pi }}]{T}_{j}({\theta }_{j};G)\}.$$

Combine (2.1) and (2.5) to verify that

2.8 $$\frac{f({y}_{i}|{\theta }_{i})\,g({\theta }_{i};\alpha ,\beta )}{{f}_{{\rm{LP}}}({y}_{i})}=\frac{{\pi }_{G}({\theta }_{i}|{y}_{i})}{1+{\sum }_{j}\,{\rm{LP}}[j;G,{\rm{\Pi }}]\,{{\mathbb{E}}}_{G}[{T}_{j}({{\rm{\Theta }}}_{i};G)|{y}_{i}]}.$$

Finish the proof of part (b) by replacing (2.8) into (2.7).

Part (c) is straightforward as

$${{\mathbb{E}}}_{{\rm{LP}}}[h({{\rm{\Theta }}}_{i})|{y}_{i}]=\int \,h({\theta }_{i})\,{\pi }_{{\rm{LP}}}({\theta }_{i}|{y}_{i})\,{\rm{d}}{\theta }_{i},$$

which is same as

$$\frac{\int \,h({\theta }_{i}){\pi }_{G}({\theta }_{i}|{y}_{i})\,\{1+{\sum }_{j}\,{\rm{LP}}[j;G,{\rm{\Pi }}]{T}_{j}({\theta }_{j};G)\}\,{\rm{d}}{\theta }_{i}}{1+{\sum }_{j}\,{\rm{LP}}[j;G,{\rm{\Pi }}]\,{{\mathbb{E}}}_{G}[{T}_{j}({{\rm{\Theta }}}_{i};G)|{y}_{i}]},$$

by (2.2). Hence, result (2.3) is immediate.

Our LP-Bayes recipe (2.1)–(2.3), admits some interesting overall structure: The usual ‘parametric’ answer multiplied by a correction factor involving LP[*j*;*G*,Π]’s. This decoupling pays dividends for theoretical interpretation as well as computation.

### Algorithm

The critical parameters of our DS(*G*, *m*) model are the LP-Fourier coefficients, which, as is evident from (1.3), could be estimated simply by their empirical counterpart $\widehat{{\rm{LP}}}[j;G,{\rm{\Pi }}]={k}^{-1}\,{\sum }_{i=1}^{k}\,{T}_{j}({\theta }_{i};G)$. But as we pointed out earlier, *θ*_1_, …, *θ*_*k*_ are unobservable. How can we then estimate those parameters? While the *θ*_*i*_’s are *unseen*, it is interesting to note that they have left their footprints in the observables *y*_1_, …, *y*_*k*_ with distribution $f({y}_{i})=\int \,f({y}_{i}|{\theta }_{i})\pi ({\theta }_{i})\,{\rm{d}}{\theta }_{i}$. Following the spirit of the EM-algorithm, an obvious proxy for *T*_*j*_(*θ*_*i*_; *G*) would be its posterior mean ${{\mathbb{E}}}_{{\rm{LP}}}[{T}_{j}({{\rm{\Theta }}}_{i};G)|{y}_{i}]$, which also naturally arises in the expression (2.1). This leads to the following ‘ghost’ LP-estimates:

2.9 $$\tilde{{\rm{LP}}}[j;G,{\rm{\Pi }}]={k}^{-1}\,\sum _{i=1}^{k}\,{{\mathbb{E}}}_{{\rm{LP}}}[{T}_{j}({{\rm{\Theta }}}_{i};G)|{y}_{i}],$$

satisfying ${\mathbb{E}}\{\tilde{{\rm{LP}}}[j;G,{\rm{\Pi }}]\}=\tilde{{\rm{LP}}}[j;G,{\rm{\Pi }}]\,(j=1\ldots ,m)$, by virtue of the law of iterated expectations. These estimates can then be refined via iterations. The following algorithm implements this strategy.

**Type-II:**
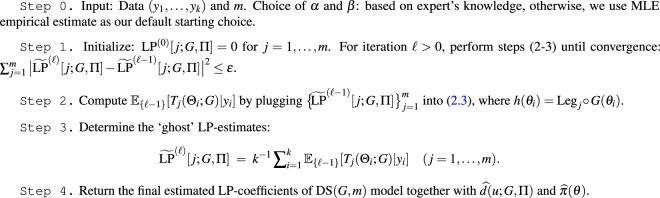
**Method of Moments: Estimation of LP-Coefficients in DS**(***G***, ***m***).

We conclude this section with a few remarks on the algorithm:

- Taking inspiration from I. J. Good’s type II maximum likelihood nomenclature^20^, we call our algorithm *Type*-*II* Method of Moments (MOM), whose computation is remarkably tractable and does not require *any* numerical optimization routine.
- To enhance the results, we smooth the output of MOM-II algorithm as follows: determine significantly non-zero LP-coefficients via Schwartz’s BIC-based smoothing. Arrange $\tilde{{\rm{LP}}}[j;G,{\rm{\Pi }}]$’s in a decreasing magnitude and choose *m* that maximizes

$${\rm{BIC}}(m)=\sum _{j=1}^{m}\,|\widehat{{\rm{LP}}}[j;G,{\rm{\Pi }}]{|}^{2}-\frac{m\,\mathrm{log}(k)}{k}.$$

See Supplementary Appendix D for more details. Furthermore, Supplementary Appendix I discusses how MOM-II Bayes algorithm can be adapted to yield LP-maximum entropy prior density estimate^21^.

### Results

In addition to the rat tumor data (cf. Section 1.2), here we introduce and analyze three additional datasets: two binomial and one Poissonian example.

- The surgical node data^22^ involves number of malignant lymph nodes removed during intestinal surgery^22^. Each of the *k* = 844 patients underwent surgery for cancer, during which surgeons removed surrounding lymph nodes for testing. Each patient has a pair of data (*n*_*i*_, *y*_*i*_), where *n*_*i*_ represents the total nodes removed from patient *i* and *y*_*i*_ ~ Bin(*n*_*i*_, *θ*_*i*_) are the number of malignant nodes among them.
- The Navy shipyard data^23^ consists of *k* = 5 samples of the number of defects *y*_*i*_ found in *n*_*i*_ = 5 lots of welding material.
- The insurance data^24^, shown in Table 4, provides a single year of claims data for an automobile insurance company in Europe^24^. The counts *y*_*i*_ ~ Poisson(*θ*_*i*_) represent the total number of people who had *i* claims in a single year.

**Figure 2:**
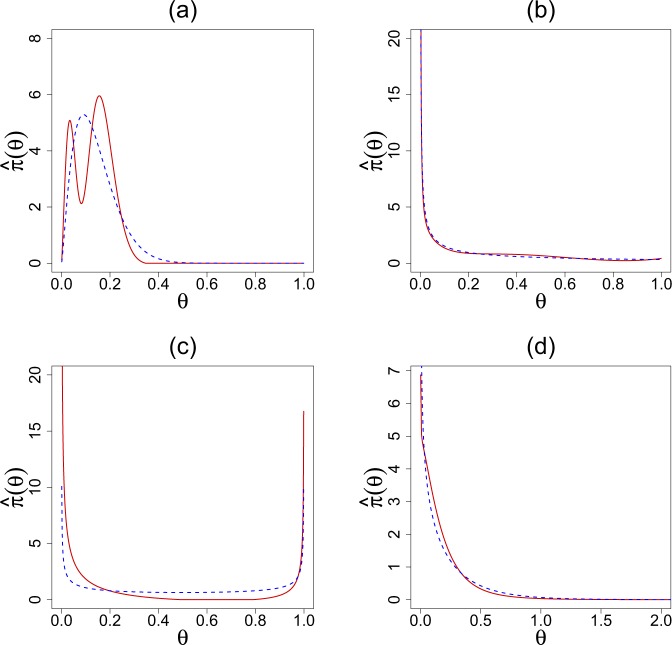
Comparisons of the DS(*G*, *m*) prior $\hat{\pi }(\theta )$ (solid red) with the respective parametric EB (PEB) priors *g*(*θ*; *α*, *β*) (dashed blue) for the (**a**) rat tumor data, (**b**) surgical node data, (**c**) Navy shipyard data, and (**d**) insurance data.

Figure 2 displays the estimated LP-DS(*G*, *m*) priors along with the default parametric (empirical Bayes) counterparts. The estimated LP-Fourier coefficients together with the choices of hyperparameters (*α*, *β*) are summarized below:

- Rat tumor data, *g* is the beta distribution with MLE *α* = 2.30, *β* = 14.08:
  2.10 $$\hat{\pi }(\theta )=g(\theta ;\alpha ,\beta )\,[1-0.50{T}_{3}(\theta ;G)].$$
- Surgical node data, *g* is the beta distribution with MLE *α* = 0.32, *β* = 1.00:
  2.11 $$\hat{\pi }(\theta )=g(\theta ;\alpha ,\beta )\,[1-0.07{T}_{3}(\theta ;G)-0.11{T}_{4}(\theta ;G)+0.09{T}_{5}(\theta ;G)+0.13{T}_{7}(\theta ;G)].$$
- Navy shipyard data, *g* is the Jeffreys prior with *α* = 0.5, *β* = 0.5:
  2.12 $$\hat{\pi }(\theta )=g(\theta ;\alpha ,\beta )\,[1-0.67{T}_{1}(\theta ;G)+0.90{T}_{2}(\theta ;G)].$$
- Insurance data, *g* is the gamma distribution with MLE *α* = 0.70 and *β* = 0.31:

2.13 $$\hat{\pi }(\theta )=g(\theta ;\alpha ,\beta )\,[1-0.26{T}_{2}(\theta ;G)].$$

The rat tumor data shows a prominent bimodal shape, which should not come as a surprise in light of Fig. 1(a). For the surgical data, DS-prior puts excess mass around 0.4, which concurs with the findings of Efron [22, Sec. 4.2]. In the case of the Navy shipyard data, our analysis corrects the starting “U” shaped Jeffreys prior to make it asymmetric with an extended peak at 0. This is quite justifiable looking at the proportions in the given data: (0/5, 0/5, 0/5, 1/5, 5/5). Finally, for the insurance data, the starting gamma prior requires a second-order (dispersion parameter) correction to yield a bona-fide $\hat{\pi }$ (2.13), which makes it slightly wider in the middle with sharper peak and tail.

## Inference

### MacroInference

A single study hardly provides adequate evidence for a definitive conclusion due to the limited sample size. Thus, often the scientific interest lies in combining several *related but* (*possibly*) *heterogeneous* studies to come up with an overall macro-level inference that is more accurate and precise than the individual studies. This type of inference is a routine exercise in clinical trials and public policy research.

#### Terbinafine data analysis

**Figure 3:**
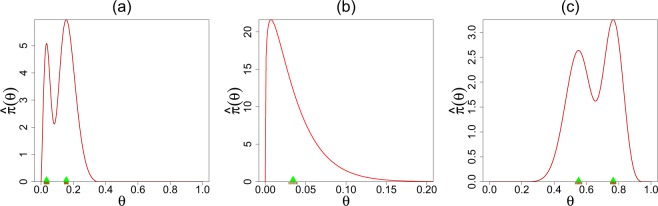
Estimated macro-inference summary along with standard errors (using smooth bootstrap) are shown. Panel (a) displays the rat tumor data modes located at 0.034 (±0.016) and 0.156 (±0.016). Panel (b) shows the estimated unimodal prior of the terbinafine data has a mean at 0.034 (±0.006). Panel (c) presents the modes of the rolling tacks data at 0.55 (±0.022) and 0.77 (±0.018).

For the terbinafine data, the aim is to combine *k* = 41 treatment arms with varying event rates and produce a pooled proportion of patients who withdrew from the study because of the adverse effects of oral anti-fungal agents. Recall that our U-function diagnostic in Fig. 1(b) indicated the parametric beta-binomial model with MLE estimates *α* = 1.24 and *β* = 34.7 as a justifiable choice for this data. Thus the adverse event probabilities across *k* = 41 studies can be summarized by the prior mean $\frac{\alpha }{\alpha +\beta }=0.034$. We apply parametric bootstrap using DS(*G*, *m*)-sampler (see Supplementary Appendix C) with *m* = 0 to compute the standard error (SE): 0.034 ± 0.006, highlighted in the Fig. 3(b). If one assumes a *single* binomial distribution for all the groups (i.e., under homogeneity), then the ‘naive’ average ${\sum }_{i=1}^{k}\,{y}_{i}/{\sum }_{i=1}^{k}\,{n}_{i}$ would lead to an overoptimistic biased estimate 0.037 ± 0.0034. In this example, heterogeneity arises due to overdispersion among the exchangeable studies. But there could be other ways too. An example is given in the following case study.

#### Rat tumor and rolling tacks data analysis

Can we always extract a “single” overall number to aptly describe *k* parallel studies? Not true, in general. In order to appreciate this, let us look at Fig. 3(a,c), which depict the estimated DS-prior for the rat tumor and rolling tacks data. We highlight two key observations:

1. *Mixed population*. The bimodality indicates the existence of two distinct groups of *θ*_*i*_’s. We call this “*structured heterogeneity*,” which is in between two extremes: homogeneity and complete heterogeneity (where there is no similarity between the *θ*_*i*_’s whatsoever). The presence of two clusters for the rolling tacks data was previously detected by Jun Liu^25^. The author further noted, “Clearly, this feature is unexpected and cannot be revealed by a regular parametric hierarchical analysis using the Beta-binomial priors.” One plausible explanation for this two-group structure was attributed to the fact that the tack data were produced by two persons with some systematic difference in their flipping. On the other hand, the bimodal shape of the rat example was not previously anticipated^14,26,27^. The resulting two groups of rat tumor experiments are enumerated in the Table 2. Although we do not have the necessary biomedical background to scientifically justify this new discovery, we are aware that potentially numerous factors (e.g., experimental design, underlying conditions, selection of specific groups of female rats) may contribute to creating this systemic variation.

**Table 2:** Two group partitions of the rat tumor studies based on K-means clustering on the posterior mode predictions (see Section 3.3 and Fig. 5(c)).

Group	Studies
1	(0, 20), (0, 20), (0, 20), (0, 20), (0, 20), (0, 20), (0, 20), (0, 19), (0, 19), (0, 19), (0, 19) (0, 18), (0, 18), (0, 17), (1, 20), (1, 20), (1, 20), (1, 20), (1, 19), (1, 19), (1, 18), (1, 18)
2	(3, 27), (2, 25), (2, 24), (2, 23), (2, 20), (2, 20), (2, 20), (2, 20), (2, 20), (2, 20), (1, 10) (5, 49), (2, 19), (5, 46), (2, 17), (7, 49), (7, 47), (3, 20), (3, 20), (2, 13), (9, 48), (10, 50) (4, 20), (4, 20), (4, 20), (4, 20), (4, 20), (4, 20), (4, 20), (10, 48), (4, 19), (4, 19), (4, 19) (5, 22), (11, 46), (12, 49), (5, 20), (5, 20), (6, 23), (5, 19), (6, 22), (6, 20), (6, 20), (6, 20) (16, 52), (15, 46), (15, 47), (9, 24)
2. *From single mean to multiple modes*. An attempt to combine the two subpopulations using a single prior mean (as carried out for the terbinafine example) would result in overestimating one group and underestimating another. We prefer *modes* of $\widehat{\pi }(\theta )$, along with their SEs, as a good representative summary, which can be easily computed by the nonparametric smooth bootstrap via DS(*G*, *m*) sampler.

Learning from big heterogeneous studies is one of the most important yet unsettled matters of modern macroinference^18,28^. Our key insight is the realization that the ‘science of combining’ critically depends on the *shape* of the estimated prior. One interesting and commonly encountered case is multimodal structure of the learned prior. In such situations, instead of the prior-mean summary, we recommend group-specific modes. Our algorithm is also capable of finding data-driven clusters of the partially exchangeable studies in a fully automated manner.

### Learning From Uncertain Data

An important problem of measurement science that routinely appears in metrology, chemistry, physics, biology, and engineering can be stated as follows: measurements are made by *k* different laboratories in the form of *y*_1_, …, *y*_*k*_ along with their estimated standard errors *s*_1_, …, *s*_*k*_. Given this uncertain data, a fundamental problem of interest is inference concerning: (i) estimation of the consensus value of the measurand, and (ii) evaluation of the associated uncertainty. The data in Table 3 are an example of such an inter-laboratory study involving *k* = 28 measurements for the level of arsenic in oyster tissue. The study was part of the National Oceanic and Atmospheric Administration’s National Status and Trends Program Ninth Round Intercomparison Exercise^29^.

**Table 3:** Measurements (sorted) along with their uncertainty from different laboratories in arsenic data.

Laboratory	1	2	3	4	5	${\boldsymbol{\cdots }}$	25	26	27	28
Measurement (*y*_*i*_)	9.78	10.18	10.35	11.60	12.01	$\cdots$	14.70	15.00	15.10	15.50
Uncertainty (*s*_*i*_)	0.30	0.46	0.07	0.78	2.62	$\cdots$	0.30	1.00	0.20	1.60

#### Arsenic data analysis

**Figure 4:**
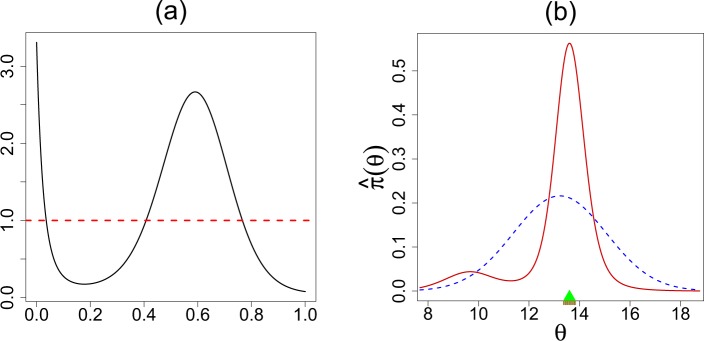
Panel (a) shows the U-function, while panel (b) compares the DS-prior $\hat{\pi }(\theta )$ (solid red) with the PEB prior *g*(*θ*; *α*, *β*) (dashed blue) for the arsenic data. Based on the estimated macro-inference summary along with standard errors (using smooth bootstrap), the best consensus value is the mode 13.6 (±0.242).

We start with the DS-measurement model: ${Y}_{i}|{{\rm{\Theta }}}_{i}={\theta }_{i}\sim {\mathscr{N}}({\theta }_{i},{s}_{i}^{2})$ and Θ_*i*_ ~ DS(*G*, *m*) (*i* = 1, …, 28) with *G* being ${\mathscr{N}}(\mu ,{\tau }^{2})$. The shape of the estimated U-function in Fig. 4(a) indicates that the pre-selected prior ${\mathscr{N}}(\hat{\mu }=13.22,{\hat{\tau }}^{2}={1.85}^{2})$ is clearly unacceptable for arsenic data, thereby disqualifying the classical Gaussian random effects model^30^. The DS-corrected $\widehat{\pi }$ shows some interesting asymmetric pattern with two-bumps. The left-mode represents measurements from three laboratories that are unlike the majority. The result of our macro-inference is shown in Fig. 4(b), which delivers the consensus value 13.6 ± 0.24. This is clearly far more resistant to fairly extreme low measurements and surprisingly, also more accurate when compared to the parametric EB estimate 13.22 ± 0.26. Most importantly, our scheme provides an automated solution to the fundamental problem of *which* (*as well as how*) measurements from the participating laboratories should be combined to form a best consensus value. Possolo^31^ fits a Bayesian hierarchical model with prior as Student’s *t*_*ν*_, where the degrees of freedom was also treated as a random variable over some arbitrary range {3, …, 118}. Although a heavy-tailed Student’s t-distribution is a good choice to ‘robustify’ the analysis, it fails to capture the inherent asymmetry and the finer modal structure on the left. Distinguishing long-tail from bimodality is an important problem of applied statistics by itself.

To summarize, there are several attractive features of our general approach: (i) it adapts to the structure of the data, yet (ii) allows the use of expert opinion to go from knowledge-based prior to statistical prior; (iii) if multiple expert opinions are available, one can also use the U-diagnostic for reconciliation–exploratory uncertainty assessment; (iv) it avoids the questionable exercise of detecting and discarding apparently unusual measurements^32^, and finally (v) our theory is still applicable for very small number of parallel cases (cf. Fig. 2(c)), a situation which is not uncommon in inter-laboratory studies.

### MicroInference

The objective of microinference is to estimate a specific microlevel *θ*_*i*_ given *y*_*i*_. Consider the rat tumor example where, along with earlier *k* = 70 studies, we have an additional current experimental data, that shows *y*_71_ = 4 out of *n*_71_ = 14 rats developed tumors. How can we estimate the probability of a tumor for this new clinical study? There could be at least three ways to answer this question:

- Frequentist MLE estimate: An obvious estimate would be the sample proportion ${\tilde{\theta }}_{i}:{y}_{71}/{n}_{71}=0.286$. This operates in an isolated manner, completely ˇ˅∨∨ignoring the additional historical information of *k* = 70 studies.
- Parametric empirical Bayes estimate: It is reasonable to expect that the historical data from earlier studies may be related to the current 71st study, thus borrowing information can result in improved estimator of *θ*_71_. Bayes posterior mean estimate ${\check{{\theta }}}_{i}={{\mathbb{E}}}_{G}[{{\rm{\Theta }}}_{i}|{y}_{i}]$ operationalizes this heuristic, which in the Binomial case takes the following form:
  31 $${\check{\theta }}_{i}=\frac{{n}_{i}}{\alpha +\beta +{n}_{i}}{\tilde{\theta }}_{i}+\frac{\alpha +\beta }{\alpha +\beta +{n}_{i}}{{\mathbb{E}}}_{G}[{\rm{\Theta }}].$$
  This is famously known as Stein’s shrinkage formula^33,34^, as it pulls the sample proportions toward the *overall* mean of the prior $\frac{\alpha }{\alpha +\beta }$. For smaller (*n*_*i*_) studies, shrinkage intensity is higher, which allows them to learn from other experiments.
- Nonparametric Elastic-Bayes estimate: Is it a wise strategy to shrink all ${\tilde{\theta }}_{i}$’s toward the grand mean 0.14? Interestingly, this shrinking point is near the valley between the twin-peaks of the rat tumor prior density estimate (verify from Fig. 3(a)) and therefore may not represent a preferred location. Then, *where to shrink?* Ideally, we want to learn only from the *relevant* subset of the full dataset–*selective shrinkage*, e.g., for rat data, it would be the group 2 of Table 2. This brings us to the question: how to rectify the parametric empirical Bayes estimate ${\check{{\theta }}}_{i}$? The formula (2.3) gives us the required (nonlinear) adjusting factor:

32 $${\hat{\theta }}_{i}=\frac{{\check{{\theta }}}_{i}+{\sum }_{j}\,\widehat{{\rm{LP}}}[j;G,{\rm{\Pi }}]\,{{\mathbb{E}}}_{G}[{{\rm{\Theta }}}_{i}{T}_{j}({{\rm{\Theta }}}_{i};G)|{y}_{i}]}{1+{\sum }_{j}\,\widehat{{\rm{LP}}}[j;G,{\rm{\Pi }}]\,{{\mathbb{E}}}_{G}[{T}_{j}({{\rm{\Theta }}}_{i};G)|{y}_{i}]},$$

dictating the magnitude and direction of shrinkage in a completely data-driven manner via LP-Fourier coefficients. Note that when $d\equiv 1$, i.e., all the LP[*j*; *G*, Π] are zero, (3.2) reproduces the parametric ${\check{{\theta }}}_{i}$. Due to its flexibility and adaptability, we call this the Elastic-Bayes estimate. This can be considered as a nonparametric class of shrinkage estimators that starts with the classical Stein’s formula and rectifies it by looking at the data.

#### Rat tumor example

**Figure 5:**
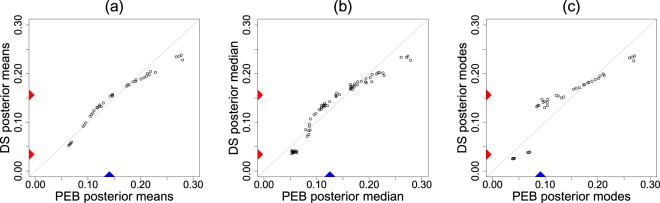
Comparisons of DS Elastic-Bayes and PEB posterior predictions of the rat tumor data: (**a**) posterior means, (**b**) posterior medians, and (**c**) posterior modes. The vertical red triangles indicate the location of the modes on the DS prior; the blue triangles respectively denote the mean, median, and mode of the parametric ${\rm{Beta}}(\hat{\alpha }=2.3,\,\hat{\beta }=14.08)$.

Figure 5 compares Stein’s empirical Bayes estimate with our Elastic-Bayes estimate for the all *k* = 70 tumor rates. Posterior mean, median, and mode of *θ*_*j*_’s are shown side by side in three plots. The departure from the 45° reference line is a consequence of “adaptive shrinkage.” Elastic-Bayes automatically shrinks the empirical ${\tilde{\theta }}_{i}$ towards the representative modes (0.034 and 0.156), whereas the Stein’s PEB estimate uses the grand mean (≈0.14) as the shrinking target for *all* the tumor rates. This is particularly prominent in Fig. 5(c) for maximum a posteriori (MAP) estimates. As before, for heterogeneous population, we prescribe posterior mode as the final prediction.

#### The Pharma-example

Our DS Elastic-Bayes estimate is especially powerful in the presence of prior-data conflict. To illustrate this point, we report a small simulation study. The goal is to compare MSE for frequentist MLE, parametric empirical Bayes, and nonparametric Elastic-Bayes estimates for a new study *y*_new_ in various levels of prior-data conflict. To capture the prior-data conflict, we consider the following model for *π*(*θ*) and *y*_new_:

$$\begin{array}{rcl}\pi (\theta ) & = & \eta \,{\rm{Beta}}(5,45)+(1-\eta )\,{\rm{Beta}}(30,70)\\ {y}_{{\rm{new}}} & \sim  & {\rm{Bin}}(50,0.3).\end{array}$$

**Figure 6:**
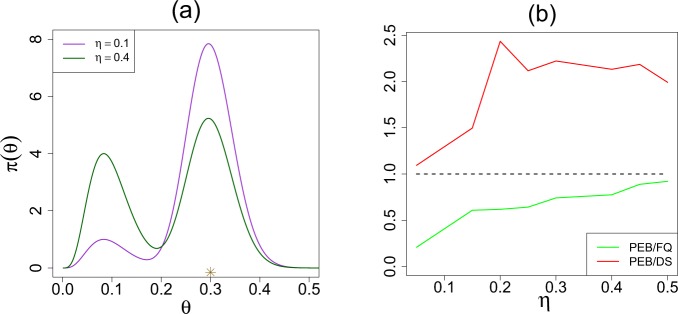
Panel (a) illustrates the prior-data conflict for *η* = 0.1 versus *η* = 0.4; ‘*’ denotes 0.3, the true mean of *y*_new_. Panel (b) shows the MSE ratios for PEB to Frequentist MLE (PEB/FQ; green) and PEB to DS (PEB/DS; red) with respect to *η*. Notice that as more prior-data conflict is introduced, DS outperforms PEB while frequentist MLE performance improves.

The parameter *η* varies from 0 to 0.50 in increments of 0.05; as *η* increases we introduce more heterogeneity into the true prior distribution and exacerbate the prior-data conflict between *π*(*θ*) and *y*_new_; see Fig. 6(a). We simulated *k* = 100 *θ*_*i*_ from *π*(*θ*), with which we generate ${y}_{i}|{\theta }_{i}\sim {\rm{Bin}}(60,{\theta }_{i})$. Using the Type-II MoM algorithm on the simulated data set, we found $\hat{\pi }$. After generating *y*_new_, we then determined the frequentist MLE, parametric EB (PEB), and the nonparametric elastic Bayes estimates of the mode. For each value of *η*, we repeated this process 250 times and found the mean squared error (MSE) for each estimate. To better illustrate the impact of prior-data conflicts, we used ratio of PEB MSE to frequentist MSE and PEB MSE to DS MSE. The results are shown in Fig. 6(b).

The Elastic-Bayes estimate outperforms the Stein’s estimate for all *η*. More importantly the efficiency of our estimate continues to increase with the heterogeneity. This is happening because elastic Bayes performs *selective* shrinkage of sample proportion towards the appropriate mode (near 0.3) and thus gains “strength” by combining information from ‘similar’ studies even when the contamination in the study population increases. An interesting observation is the performance of the frequentist MLE estimate; as the data becomes more heterogeneous, the frequentist MLE shows improvement with respect to the Stein’s PEB estimate. Our simulation depicts a scenario that is very common in historic-controlled clinical trials, where the heterogeneity arises due to changing conditions. Additional comparisons with other empirical Bayes procedures can be found in Supplementary Appendix G.

#### Three additional real examples

**Figure 7:**
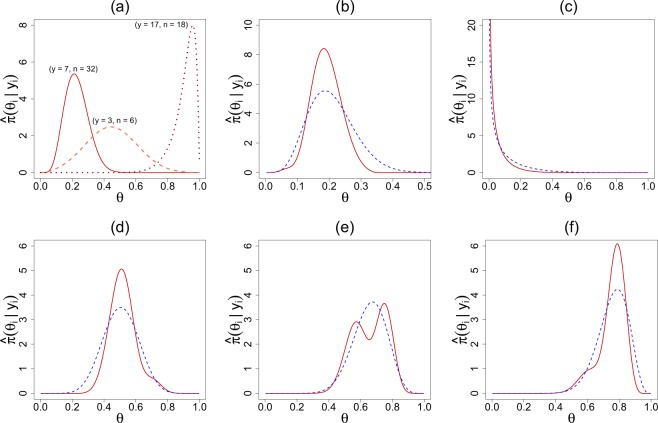
Panel (a) shows DS posterior plots of three observations from the surgical node data: (*y* = 7, *n* = 32), (*y* = 3, *n* = 6), and (*y* = 17, *n* = 18). For panels (b) through (f), red denotes the DS posterior and blue dashed is the PEB posterior. Panel (b) is $\hat{\pi }({\theta }_{71}|{y}_{71}=4)$ for the rat tumor data. Panel (c) displays $\hat{\pi }({\theta }_{6}|{y}_{6}=0)$ for the Navy shipyard data. The second row shows the posterior distributions of (**d**) *y*_*i*_ = 3, (**e**) *y*_*i*_ = 6, and (**f**) *y*_*i*_ = 8 from the rolling tacks data.

Figure 7 shows the posterior plots for specific studies in four of our data sets: surgical node, rat tumor, Navy shipyard, and rolling tacks. In studies like the surgical node data, personalized predictions are typically valuable. Figure 7(a) shows posterior distributions for three selected patients, which are indistinguishable from Efron’s deconvolution answer^35^ [Fig. 4]; the patient with *n*_*i*_ = 32 and *y*_*i*_ = 7 shows almost certainly *θ*_*i*_ > 0.5, i.e., he or she is highly prone to positive lymph nodes, and thus should be referred to follow-up therapy. With regard to the rat tumor data, Fig. 7(b) depicts the DS-posterior distribution of *θ*_71_ along with its parametric counterpart *π*_*G*_(*θ*_71_|*y*_71_, *n*_71_). Interestingly, the DS nonparametric posterior shows less variability; this possibly has to do with the selective learning ability of our method, which learns from similar studies (e.g. group 2), rather than the whole heterogeneous mix of studies. We see similar phenomena in the rolling tacks data, where panel (d): *y*_*i*_ = 3, is more reflective of the first mode and panel (f): *y*_*i*_ = 8, of the second. Panel (e) shows the bimodal posterior for *y*_*i*_ = 6 case. Finally, the Navy shipyard data (Fig. 7(c)) exhibits another advantage of DS priors: it works equally well for small *k*. The DS-posterior mean estimate for *y*_6_ = 0 is 0.0471, which is consistent with the findings of Sivaganesan and Berger^36^ [p. 117].

### Poisson Smoothing: The Two Cultures

We consider the problem of estimating a vector of Poisson intensity parameters *θ* = (*θ*_1_, …, *θ*_*k*_) from a sample of *Y*_*i*_|*θ*_*i*_ ~ Poisson(*θ*_*i*_), where the Bayes estimate is given by:

3.3 $${\mathbb{E}}[{\rm{\Theta }}|Y=y]=\frac{{\int }_{0}^{\infty }\,\theta [{e}^{-\theta }\,{\theta }^{y}/y!]\,\pi (\theta )\,{\rm{d}}\theta }{{\int }_{0}^{\infty }\,[{e}^{-\theta }\,{\theta }^{y}/y!]\,\pi (\theta )\,{\rm{d}}\theta };\,y=0,1,2,\ldots .$$

Two primary approaches for estimating (3.3):

- Parametric Culture^37,38^: If one assumes *π*(*θ*) to be the parametric conjugate Gamma distribution $g(\theta ;\alpha ,\beta )=\frac{1}{{\beta }^{\alpha }{\rm{\Gamma }}(\alpha )}{\theta }^{\alpha -1}{e}^{-\theta /\beta }$, then it is straightforward to show that Stein’s estimate takes the following analytical form ${\check{{\theta }}}_{i}=\frac{{y}_{i}+\alpha }{{\beta }^{-1}+1}$, weighted average of the MLE *y*_*i*_ and the prior mean *αβ*.
- Nonparametric Culture^4,7,39^: This was born out of Herbert Robbins’ ingenious observation that (3.3) can alternatively be written in terms of marginal distribution $(y+1)\frac{f(y+1)}{f(y)}$, and thus can be estimated non-parametrically by substituting empirical frequencies. This remarkable “prior-free” representation, however, does not hold in general for other distributions. As a result, there is a need to develop methods that can bite the bullet and estimate the prior *π* from the data. Two such promising methods are Bayes deconvolution^7^ and the Kiefer-Wolfowitz non-parametric MLE (NPMLE)^39,40^. Efron’s technique can be viewed as *smooth* nonparametric approach, whereas NPMLE generates a discrete (atomic) probability measure. For more discussion, see Supplementary Appendix A2.

#### The Third Culture

Each EB modeling culture has its own strengths and shortcomings. For example, PEB methods are extremely efficient when the true prior is Gamma. On the other hand, the NEB methods possess extraordinary robustness in the face of a misspecified prior yet they are inefficient when in fact $\pi \equiv {\rm{Gamma}}(\alpha ,\beta )$. Noticing this trade-off, Robbins raised the following intriguing question^10^: *how can this efficiency*-*robustness dilemma be resolved in a logical manner?* To address this issue, we must design a data analysis protocol that offers a mechanism to answer the following *intermediate* modeling questions (before jumping to estimate $\hat{\pi }$): Can we assess whether or not a Gamma-prior is adequate in light of the sample-information? In the event of a prior-data conflict, how can we estimate the ‘missing shape’ in a completely data-driven manner? All of these questions are at the heart of our ‘Bayes *via* goodness-of-fit’ formulation, whose goal is to develop a third culture of generalized empirical Bayes (gEB) modeling by uniting the parametric and non-parametric philosophies. Compute the DS Elastic-Bayes estimate by substituting ${\check{{\theta }}}_{i}=\frac{{y}_{i}+\alpha }{{\beta }^{-1}+1}$ in the Eq. (3.2), which reduces to the PEB answer when $d(u;G,{\rm{\Pi }})\equiv 1$ (i.e, the true prior is a Gamma) and modifies non-parametrically, only when needed; thereby turning Robbins’ vision into action (see Supplementary Appendices A and G for more discussions on this point).

#### The insurance data

Table 4 reports the Bayes estimates ${\mathbb{E}}[\theta |Y=y]$ for the insurance data. We compare five methods: parametric Gamma, classical Robbins’ EB, Efron’s Deconvolve, Koenker’s NPMLE, and our procedure. The raw-nonparametric Robbins’ estimator is clearly erratic at the tail due to data-sparsity. The PEB estimate overcomes this limitation and produces a stable estimate; but *is it dependable?* Should we stop here and report this as our final result? Our exploratory U-diagnostic tells that (consult Sec. 2.3) the PEB estimate needs a second-order correction to resolve the discrepancy between the Gamma prior and data. The improved LP-nonparametric Stein estimates are shown in the last row of Table 4.

**Table 4:** For the insurance data set, estimates for the number of claims expected in the following year by an individual who made *y* claims during the present year, $\hat{{\mathbb{E}}}(\theta |Y=y)$, by five different methods.

Claims *y*	0	1	2	3	4	5	6	7
Counts	7840	1317	239	42	14	4	4	1
Gamma PEB	0.164	0.398	0.633	0.87	1.10	1.34	1.57	1.80
Robbins’ EB	0.168	0.363	0.527	1.33	1.43	6.00	1.75	—
Deconvolve	0.164	0.377	0.642	1.14	2.13	3.45	4.47	5.08
NPMLE	0.168	0.362	0.534	1.24	2.21	2.53	2.58	2.58
DS Elastic-Bayes	0.156	0.322	0.517	0.744	1.02	1.56	3.01	5.24

#### The butterfly data

**Figure 8:**
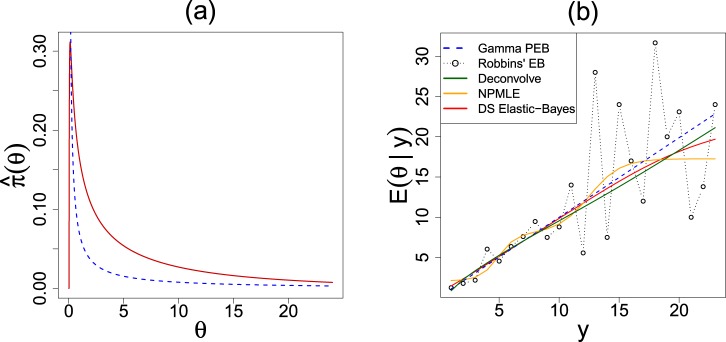
Panel (a) displays the estimated DS(*G*, *m* = 4) prior (solid red) with the PEB Gamma prior *g*(*θ*; *α*, *β*) (dashed blue) for the butterfly data; these results indicate that Fisher’s Gamma-prior guess required some correction. Panel (b) shows estimates for the number of butterfly species caught in the following year $\hat{{\mathbb{E}}}(\theta |x)$ by the Gamma PEB, Robbins’ formula, Bayesian deconvolution, NPMLE, and our Elastic-Bayes estimate.

The next example is Corbet’s Butterfly data^37^ – one of the earliest examples of empirical Bayes. Alexander Corbet, a British naturalist, spent two years in Malaysia trapping butterflies in the 1940s. The data consist of the number of species trapped exactly *y* times in those two years for *y* = 1, …, 24. Figure 8(b) plots different Bayes estimates. The Robbins’ procedure suffers from similar ‘jumpiness.’ The blue dotted line represents the linear PEB estimate with *α* = 0.104 and *β* = 89.79 (same as of Efron and Hastie^24^, Eq. 6.24) estimated from the zero-truncated negative binomial marginals. Our DS-estimate is almost sandwiched between the PEB and Deconvolve answer. The NPMLE method (the orange curve) yields some strange looking sinusoidal pattern, probably due to overfitting. In conclusion, we must say that the triumph of our procedure as compared to the other Bayes estimators lies in its automatic adaptability that Robbins alluded in his 1980 article^10^.

## Discussions

We laid out a new mechanics of data modeling that effectively consolidates Bayes and frequentist, parametric and nonparametric, subjective and objective, quantile and information-theoretic philosophies. However, at a practical level, the main attractions of our “Bayes *via* goodness-of-fit” framework lie in its (i) ability to quantify and protect against prior-data conflict using exploratory graphical diagnostics; (ii) theoretical simplicity that lends itself to analytic closed-form solutions, avoiding computationally intensive techniques such as MCMC or variational methods.

We have developed the concepts and principles progressively through a range of examples, spanning application areas such as clinical trials, metrology, insurance, medicine, and ecology, highlighting the core of our approach that gracefully combines Bayesian way of thinking (parameter probability where prior knowledge can be encoded) with a frequentist way of computing via goodness-of-fit (evaluation and synthesis of the prior distribution). If our efforts can help to make Bayesian modeling more attractive and transparent for practicing statisticians (especially non-Bayesians) by even a tiny fraction, we will consider it a success.

### Data availability

All datasets and the computing codes are available via free and open source R-software package BayesGOF. The online link: https://CRAN.R-project.org/package=BayesGOF.

## Electronic supplementary material

Supplementary Material

## References

[1] Efron B (1986). Why isn’t everyone a Bayesian?. The Am. Stat..

[2] Sims, C. Understanding non-Bayesians. *Unpubl*. *chapter*, *Dep*. *Econ*. *Princet*. *Univ*. (2010).

[3] Stigler SM (1982). Thomas Bayes’s Bayesian inference. J. Royal Stat. Soc. Ser. A (General).

[4] Robbins, H. An empirical Bayes approach to statistics. In *Proceedings of the Third Berkeley Symposium on Mathematical Statistics and Probability*, *Volume 1*: *Contributions to the Theory of Statistics*, 157–164 (1956).

[5] Good I (1992). The Bayes/non-Bayes compromise: A brief review. J. Am. Stat. Assoc..

[6] Rubin DB (1984). Bayesianly justifiable and relevant frequency calculations for the applied statistician. The Annals Stat..

[7] Efron B (2003). Robbins, empirical Bayes and microarrays. The Annals Stat..

[8] Dempster AP (1975). A subjectivist look at robustness. Bull. Intern. Stat. Inst.

[9] Berger JO (1994). An overview of robust Bayesian analysis (with discussion). Test.

[10] Robbins H (1980). An empirical Bayes estimation problem. Proc. Natl. Acad. Sci..

[11] Mukhopadhyay, S. & Parzen, E. LP approach to statistical modeling. *arXiv preprint arXiv*:*1405*.*2601* (2014).

[12] Good IJ (1983). The philosophy of exploratory data analysis. Philos. science.

[13] Gelman A, Simpson D, Betancourt M (2017). The prior can often only be understood in the context of the likelihood. Entropy.

[14] Gelman, A. *et al*. *Bayesian Data Analysis*, Third Edition. Chapman & Hall/CRC Texts in Statistical Science (Taylor & Francis, 2013).

[15] Young-Xu Y, Chan KA (2008). Pooling overdispersed binomial data to estimate event rate. BMC Med. Res. Methodol..

[16] Beckett L, Diaconis P (1994). Spectral analysis for discrete longitudinal data. Adv. Math..

[17] Sacks HS, Chalmers TC, Blum AL, Berrier J, Pagano D (1990). Endoscopic hemostasis: an effective therapy for bleeding peptic ulcers. J. Am. Med. Assoc..

[18] Efron B (1996). Empirical Bayes methods for combining likelihoods. J. Am. Stat. Assoc..

[19] Gelman, A., Meng, X.-L. & Stern, H. Posterior predictive assessment of model fitness via realized discrepancies. *Stat*. *Sinica* 733–760 (1996).

[20] Good IJ (1983). Good thinking: The foundations of probability and its applications.

[21] Mukhopadhyay S (2017). Large-scale mode identification and data-driven sciences. Electron. J. Stat..

[22] Efron B (2016). Empirical Bayes deconvolution estimates. Biom..

[23] Martz H, Lian M (1974). Empirical bayes estimation of the binomial parameter. Biom..

[24] Efron, B. & Hastie, T. *Computer Age Statistical Inference*, vol. 5 (Cambridge University Press, 2016).

[25] Liu, J. S. Nonparametric hierarchical Bayes via sequential imputations. *The Annals Stat*. 911–930 (1996).

[26] Tarone RE (1982). The use of historical control information in testing for a trend in proportions. Biom..

[27] Dempster AP, Selwyn MR, Weeks BJ (1983). Combining historical and randomized controls for assessing trends in proportions. J. Am. Stat. Assoc..

[28] Cox DR (1990). Comment: The 1988 Wald Memorial Lectures: The present position in Bayesian statistics. Stat. Sci..

[29] Willie, S. & Berman, S. Ninth round intercomparison for trace metals in marine sediments and biological tissues. *NRC*/*NOAA* (1995).

[30] Rukhin AL, Vangel MG (1998). Estimation of a common mean and weighted means statistics. J. Am. Stat. Assoc..

[31] Possolo A (2013). Five examples of assessment and expression of measurement uncertainty. Appl. Stoch. Model. Bus. Ind..

[32] Toman B, Possolo A (2009). Laboratory effects models for interlaboratory comparisons. Accreditation Qual. Assur..

[33] Stein C (1955). Inadmissibility of the usual estimator for the mean of a multivariate normal distribution. Proc. Third Berkeley Symp. on Math. Stat. Probab..

[34] Efron B, Morris C (1975). Data analysis using Stein’s estimator and its generalizations. J. Am. Stat. Assoc..

[35] Cox D, Efron B (2017). Statistical thinking for 21st century scientists. Sci. Adv..

[36] Sivaganesan S, Berger J (1993). Robust Bayesian analysis of the binomial empirical Bayes problem. Can. J. Stat..

[37] Fisher, R. A., Corbet, A. S. & Williams, C. B. The relation between the number of species and the number of individuals in a random sample of an animal population. *The J*. *Animal Ecol*. 42–58 (1943).

[38] Maritz J (1969). Empirical Bayes estimation for the poisson distribution. Biom..

[39] Gu J, Koenker R (2016). On a problem of Robbins. Int. Stat. Rev..

[40] Kiefer, J. & Wolfowitz, J. Consistency of the maximum likelihood estimator in the presence of infinitely many incidental parameters. *The Annals Math*. *Stat*. 887–906 (1956).

