# Supplementary material for: Generalized Empirical Bayes Modeling via Frequentist Goodness of Fit
Source: Sci Rep. 2018 Jul 2;8:9983. doi: 10.1038/s41598-018-28130-5 (PMC6040203; doi:10.1038/s41598-018-28130-5)
Supplement: Supplementary file 1 — Supplementary Material [file 41598_2018_28130_MOESM1_ESM.pdf]

Supplementary Material for "Generalized Empirical Bayes Modeling via Frequentist Goodness of Fit"

Subhadeep Mukhopadhyay*, Douglas Fletcher

Temple University, Department of Statistical Science

Philadelphia, Pennsylvania, 19122, U.S.A.

* To whom correspondence should be addressed; E-mail: deep@temple.edu

This supplementary document contains nine Appendices, organized as follows:

- Appendix A: Connections with other Bayesian modeling cultures.
- Appendix B: More insights into the LP-basis functions.
- Appendix C: The DS(G, m) sampler.
- Appendix D: Other practical considerations.
- Appendix E: Software.
- Appendix F: Data Catalogue.
- Appendix G: The Robbins' puzzle.
- Appendix H: Example with covariates.
- Appendix I: Maximum-Entropy enhancement.

A. CONNECTIONS WITH OTHER BAYESIAN MODELING CULTURES

In this section, we explore the relationship of our approach with other existing Bayesian data modeling cultures from philosophical and computational perspective. We will show that our formulation can be interpreted from surprisingly diverse perspectives.

A1. Robust Bayesian Methods

Our view of going from a unique prior assumption to a class of priors for robust Bayesian modeling was shaped by the Jim Berger's outstanding article [1]. In the same spirit of the ϵ-contamination class [2], our U-function $d(u; G, \Pi)$ can be thought of as an automatic robustifier for standard (conjugate) priors. Thus, our approach may attain similar goals in a more computationally friendly way. Finally, we completely agree with Berger [1] that 'The major objection of non-Bayesians to Bayesian analysis is uncertainty in the prior, so eliminating this concern can make Bayesian methods considerably more appealing.'

A2. Empirical Bayes Methods

Empirical Bayes approaches use data to determine the prior. While parametric empirical Bayes [PEB] [3] fixes the hyperparameters based on the data, nonparametric empirical Bayes [NEB] [4] makes no assumptions on the prior's form and develops it based solely on the data. In particular, Brad Efron [5, 6] advocate a *smooth* nonparametric exponential family model: $\log \pi(\theta) = \sum_{j=0}^{m} \beta_j \theta^j$ for the prior distribution where $\boldsymbol{\beta} = (\beta_0, \ldots, \beta_m)$ is estimated by maximizing the marginal log-likelihood function.

Example 1. The dotted line in Figure 9(a) denotes the non-parametrically estimated Efron's $\widehat{\pi}$ based on two-dimensional sufficient vector $S = (\theta, \theta^2)$ for the ulcer data [5]. At a first glance, it appears strikingly close to the conjugate normal prior $\mathcal{N}(-1.17, 0.98)$, marked as the bold red line. Perhaps the reader may be curious to know whether '$\pi(\theta) \equiv$ PEB Normal' here? This is indeed the case, as already shown in Figure 1(b) of the main paper. Our generalized empirical Bayes (gEB) framework automatically reduces to PEB when the data is consistent with the assumed parametric prior and modifies it non-parametrically otherwise. The output of the combined inference from $k = 40$ clinical trials is shown as a green triangle -1.17 ± 0.197, which is quite close[†] to the Efron's nonparametric answer [5] -1.22 ± 0.26. The negative macro-estimate of the log-odds ratio parameters suggests that the new surgical treatment for stomach ulcers is overall more effective than the existing one.

Another attractive NEB technique is based on non-parametric maximum likelihood estimate (NPMLE): maximize the log-likelihood $\sum_{i=1}^{k} \log\left\{\int f(y_i|\theta)\ \mathrm{d}\Pi(\theta)\right\}$ over the set of all $\pi(\theta)$ on $\mathbb{R}$, which is known to be a notoriously difficult problem. Thanks to Gu and Koenker [8], an approximate NPMLE can be estimated via convex optimization technique (interior point method) instead of classical EM (Expectation-Maximization) algorithm [9], thereby making it a computationally feasible alternative.

Example 2. NPMLE imposes no structural constraint and produces an estimated prior as discrete measure supported on at most k points within the data range. Figure 9(b) shows its application to the child illness data [10], which comes from a study that followed $k = 602$ pre-school children in north-east Thailand from June 1982 through September 1985. Researchers recorded the number of times (y) a child became sick during every 2-week period. Using the DS-Bayes method, we have $\widehat{\pi}(\theta)$ where $g(\theta)$ is a gamma distribution

[†]The slight gain in accuracy for our method lies in the style of estimation that proceeds *via* goodness-of-fit. Constructing prior by validating its credibility (using frequentist criterion) may also strengthen the Bayesian objectivity that Brad Efron [7] alluded to his article "Why isn't everyone a Bayesian?"

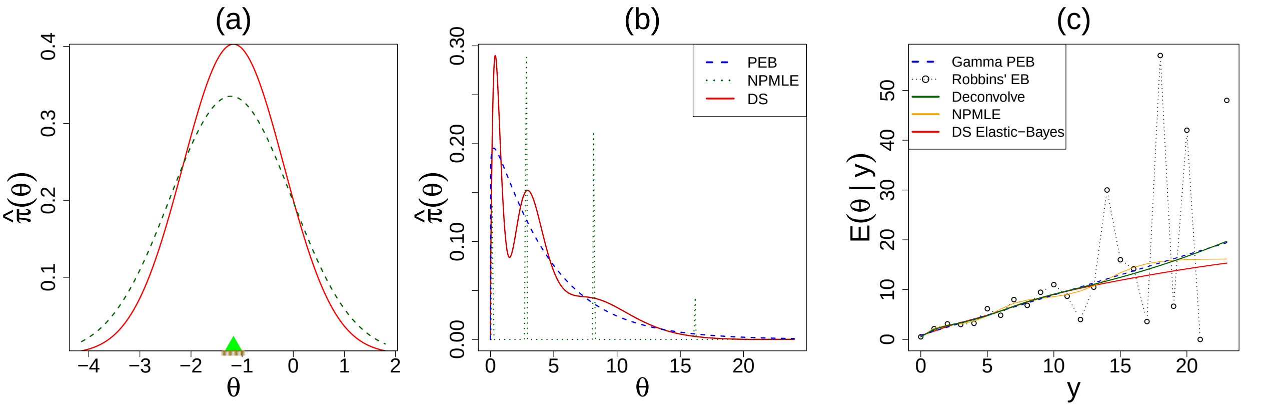

Figure 9: Comparisons of DS(G, m) (red) with other empirical Bayes modeling cultures (green): (a) The DS-estimated prior is compared with Efron's exponential prior model [5]; (b) The DS distribution for the child illness data compared to NPMLE (the dotted line); (c) Estimates for the number of illnesses in the following year $\hat{\mathbb{E}}(\theta \mid x)$ by Gamma PEB, Robbins' formula, Bayesian deconvolution, NPMLE, and our elastic-Bayes estimate.

Table 5: Run-time comparisons between DS-Bayes and two other BNP methods: Dirichlet prior (DP), and Bernstein-Dirichlet (BDP) model. All methods were run using an Intel®Core™ i5-7200 CPU @ 2.50GHz. `DPpackage` uses `C++` complier to speed-up, while ours is a prototype version implemented in `R`.

Data Set	# Studies (k)	DS Time	DP Time	Ratio DP to DS	BDP Time	Ratio BDP to DS
Rat Tumor	70	1.83	10.42	5.69	3457.75	1889.5
Surgical Node	844	30.95	189.3	6.12	45292.15	1463.4
Terbinafine	41	1.7	5.46	3.2	1883.18	1107.8
Rolling Tacks	320	8.27	59.16	7.15	16569.78	2003.6
Arsenic	28	0.47	13.09	27.8	433.29	254.9

with $\hat{\alpha} = 1.06$ and $\hat{\beta} = 4.19$ as

$$\hat{\pi}(\theta) = \text{Gamma}(\theta; \alpha, \beta)\big[1 - 0.13T_3(\theta; G) - 0.28T_6(\theta; G)\big]. \tag{1}$$

Our method produces a smooth, grid-free $\hat{\pi}$ that accurately captures the overall shape. Figure 9(c) plots the Bayes estimates $\mathbb{E}[\Theta_i|Y = y]$ for all competing methods. For Efron's `Deconvolve` we have used c0 = 2 and pDegree = 25, which seems to produce a reasonable prior density estimate for this example. A careful look at the plot reveals an 'oscillating' NPMLE Bayes estimates (orange curve), which many not be particularly desirable.

A3. Dirichlet-Process-based Approaches

Bayesian nonparametric [BNP] technique assigns prior distribution on infinite-dimensional spaces of probability models. The majority of work on Bayesian nonparametrics utilizes a Dirichlet process prior [11]. The computational cost of BNP is severe and produces prior

on a set of discrete probability measures that demands an additional layer of smoothing. Figure 10 contrasts Dirichlet-process based Beta-Binomial models [12] with our DS-Bayes model. There are few remarks warranted here:

- BNP method requires careful tuning of several hyper-priors values, which from our experience can be quite sensitive (see Figure 10). Without practical guidance, this "fishing expedition" can potentially overwhelm one who seeks to confidently use it in practice. On the contrary, our method finds practically the same answer without adjusting multiple hyper-prior values.
- The posterior inferences of BNP are highly complex and require computationally expensive MCMC. In contrast, the beauty of our approach is that it provides compact analytical expressions that make the computation much more amicable.
- The flexibility of BNP comes with the heavy task of estimating a massive number of parameters–"massively parametric Bayes." Contrast this with $\mathrm{DS}(G, m)$ model, which provides a reduced-dimensional characterization of the prior distribution with a closed form solution that is computationally efficient (see Table 5) and produces smooth estimates in one-shot. For additional comments see the 'Critical Appraisal' section.

A4. Weakly Informative Priors

A weakly informative prior [WIP] is a proper prior that intentionally provides less information than available prior knowledge. This lies somewhere between a fully subjective and a fully objective prior [13, 14].

One can also view our approach from a WIP-angle where $d(u; G, \Pi)$ acts as a "spreading/weakening function" of the subjective prior $g(\theta)$, which we *learn from the data.* In the $\mathrm{DS}(G, m)$ language: m is the radius of spread; the larger the m, the greater possibility you allow for changing the shape (the process of weakening) of the presumed scientific prior distribution $g(\theta)$. These analogies suggest that our concepts and notations might provide a systematic way to formulate the WIP philosophy by addressing the debates around "WIP is a subjective prior with ad hoc large but bounded support." This reformulation can also bring some tangible computational gain.

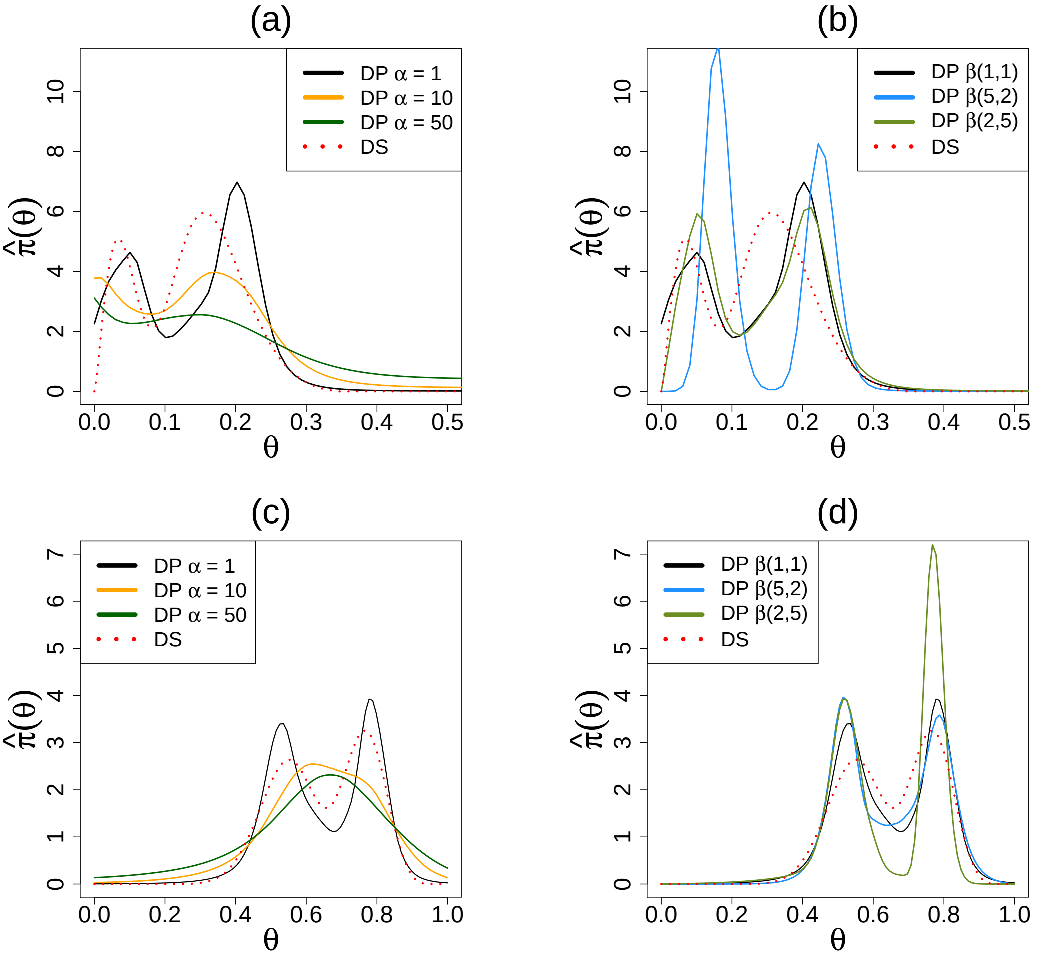

Figure 10: Illustrations of the different settings for BNP modeling with a Dirichlet process prior. Panel (a) displays results for the rat tumor data using uniform base prior while varying α. Panel (b), also for the rat tumor data, fixes $\alpha = 1$ and varies the base prior between uniform, Beta$(5, 2)$ and Beta$(2, 5)$. Panels (c) and (d) use the same settings as (a) and (b), but applied to the rolling tacks data.

A Critical Appraisal

We close this section by highlighting some of the unique aspects and practical advantages of our technique:

- *Clarifying the Motivation*: Let's start by reminding ourselves that the core motivation behind the 'Bayes *via* goodness-of-fit' is more than just another recipe for estimating the prior from data. To understand the mysterious prior in a transparent and definitive way, it is critical to ask: How can we provide automatic protection from unqualified specifications of prior distribution? How do we assess the prior-uncertainty using exploratory graphical tools? How can we prescribe a revised statistical-prior starting from the user-specified scientific-prior? As it stands, these fundamental questions are usually left unanswered in traditional Bayes framework and create a major obstacle for

non-Bayesian practitioners to confidently use Bayesian tools. Consequently, there is a need to address these issues in a formal manner to bring much-needed transparency. This paper has taken some solid steps toward this goal with a methodology that is readily usable for wide-range of applied problems. We believe that our technology can become an integral part of applied Bayesian modeling.

- *Theoretical Novelty*: Our proposed theory, which is general enough to include almost all commonly-used models, yields analytic closed-form solutions for posterior modeling. This is noteworthy for the simple reason that none of the nonparametric methods mentioned above can stand by this claim.

- *Theoretical Simplicity*: The whole 'Bayes *via* Goodness-of-fit' framework can be developed starting from a few basic principles, without requiring any exotic theoretical treatment. This could add invaluable transparency to the theory and practice of (empirical) Bayesian statistics.

- *Exploratory Side*: Our approach brings a distinct exploratory flavor into the empirical-Bayes modeling. It encourages interactive data analysis rather than blindly 'turning the crank.' Through numerous examples, we demonstrated how this mode of operation often leads to more insights into the data that are typically infeasible under a business-as-usual Bayesian modus operandi.

- *Computational Side*: Simplicity of implementation and computational ease are the two hallmarks of our method. No expensive MCMC or even sophisticated optimization routines are required! We made a sincere effort to design a practical Bayesian data analysis tool that is both simpler to comprehend and easy to implement.

- *A Third Empirical Bayes Culture.* Our empirical Bayes approach is neither parametric nor nonparametric. As argued in Section 3.4 (of the main paper), our algorithmic approach blends conventional PEB and Robbins-style full-fledged NEB. Our goal is to combine the best of both worlds, in the sense that it reduces to PEB (ulcer data example) when in fact the default parametric g is appropriate, while in the event of prior-data conflict (rat tumor or child illness data), it automatically produces reliable nonparametric procedures. And in this whole story, the U-function $d(u;G,\Pi)$ acts as the "connector" between these two extreme philosophies. Overall, we are hopeful that our Generalized EB (gEB) modeling framework might expedites the development of a new *genre* of 'unified' Bayesian algorithms [15] by leveraging the rich interplay between two extreme EB philosophies.

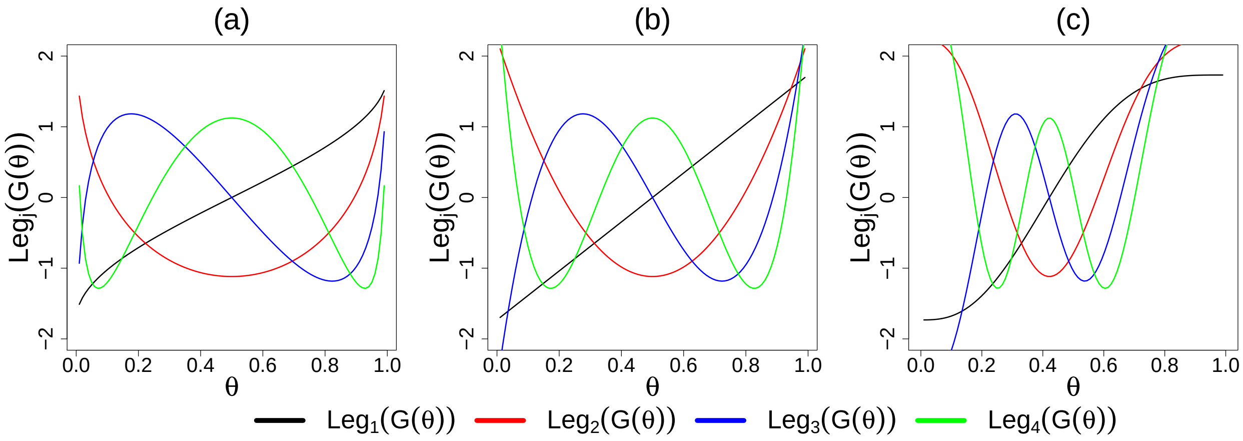

Figure 11: LP-polynomials $T_j(\theta; G_{\alpha,\beta})$ for `family= "beta"` with the following (α, β) choices: (a) Jeffrey's prior ($\alpha = \beta = 0.5$), (b) uniform prior ($\alpha = \beta = 1$), and for (c) Beta($\alpha = 3, \beta = 4$). Note that for $U[0,1]$ (the middle panel): $T_j \equiv \mathrm{Leg}_j$, as $G(\theta)$ is simply θ in this case.

B. MORE INSIGHTS INTO THE LP-BASIS FUNCTIONS

Here we will show the shapes of the LP-polynomials, focusing only the Binomial case. It works similarly for other families.

The $\{T_j(\theta; G_{\alpha,\beta})\}_{j\geqslant 1}$ denotes the class of orthonormal polynomials of the beta distribution with parameters α and β. Let $T_j(\theta; G_{\alpha,\beta}) = \mathrm{Leg}_j\{G_{\alpha,\beta}(\theta)\}$ and $G_{\alpha,\beta}(\theta) = \frac{1}{\mathbf{B}(\alpha,\beta)} \int_0^\theta \phi^{\alpha-1}(1-\phi)^{\beta-1} d\phi$. Figure 11 displays the shapes of top four LP polynomials for three different sets of parameters. We generate these polynomials with the following R code:

```
LP.basis.beta <- function(y, g.par, m){
#######################################
##  g.par: parameters for the beta distribution
#######################################
require(orthopolynom)
u <- pbeta(y, g.par[1], g.par[2]) # computes G(y)
poly <-  slegendre.polynomials(m,normalized=TRUE)
TY <- matrix(NA,length(u),m)
for(j in 1:m) TY[,j] <- predict(poly[[j+1]],u)
return(TY)}
```

C. THE DS(G, m) SAMPLER

The following algorithm generates samples from the DS(G, m) model via accept/reject scheme.

DS(G, m) Sampling Algorithm

`Step 1.` Generate Θ from g; independent of Θ, generate U from Uniform$[0, 1]$.

`Step 2.` Accept and set $\Theta^* = \Theta$ if

$$\widehat{d}[G(\theta); G, \Pi] > U \max_u \{\widehat{d}(u; G, \Pi)\};$$

otherwise, discard Θ and return to Step 1.

`Step 3.` Repeat until simulated sample of size k, $\{\theta_1^*, \theta_2^*, \cdots, \theta_k^*\}$.

Note that when $\widehat{d} \equiv 1$ then the DS(G, m) automatically samples from parametric G.

D. OTHER PRACTICAL CONSIDERATIONS

In the event that *no* prior knowledge is available, selecting the parametric conjugate prior G with empirically estimated α, β in conjunction with our Type-II Method of Moments algorithm (sec. 2.2) will provide a quick estimate of the oracle π. The algorithm finds the 'best' approximating prior model given `m.max`: the maximum complexity that the subject-matter experts want to entertain. From our experience with DS(G, m) model, we found `m.max` = 8 works satisfactorily well in practice (in fact in all our examples 8 was our default choice), which encompasses the space of reasonable priors around G. Given this maximum radius, our method generates a deviance plot, where the "elbow" shape (see Figure 12 (a)) denotes the most likely model dimension. This procedure is fully incorporated into our algorithm so that practitioners can use it in a completely automatic manner.

Illustration. Consider the model: $y_i|\theta_i \sim$ Binomial$(50, \theta_i)$ with $i = 1, \ldots, k = 90$ and the true prior distribution $\pi(\theta) = .3\text{Beta}(4, 6) + .7\text{Beta}(20, 10)$. Our goal is to see how well we can approximate the unknown π without any prior knowledge of its shape. The following R code can be used to reproduce our findings reported in Figure 12.

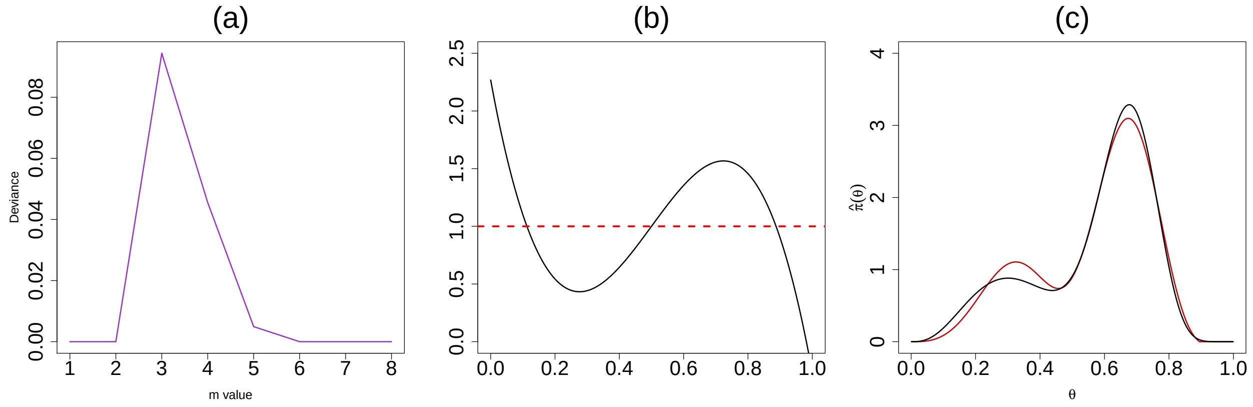

Figure 12: Analysis for simulated data based on Type-II Method of Moments algorithm. The first panel (a) finds the "elbow" in the BIC(m) deviance plot at $m = 3$; (b) shows the U-function, while (c) plots the true $\pi(\theta)$ (black) along with the estimated DS prior (red) $\hat{\pi}(\theta) = g(\theta; \hat{\alpha}, \hat{\beta})\big[1 - 0.48T_3(\theta; G)\big]$ with MLE $\hat{\alpha} = 4.16$ and $\hat{\beta} = 3.04$.

```
set.seed(8701)
k <- 90
n.i <- 50
n.vec <- rep(n.i,k)
k1 <- ceiling(.7*k)
#Test Simulation: Mixed beta Distribution
theta.sim <- c(rbeta(k1,20,10), rbeta( (k-k1),4,8))
y.sim <- sapply(theta.sim, rbinom, size = n.i, n = 1)
sim.df <- data.frame(y = y.sim, N = n.vec)
##Run Type II MoM Algorithm
sim.start <- gMLE.bb(sim.df$y,sim.df$N)$estimate
sim.LP.par <- DS.prior(sim.df, g.par = sim.start, family = "Binomial")
```

The `sim.start` object holds the MLE estimate for the initial parameters for G. From the `sim.LP.par` object, we generate diagnostic and analysis plots for appropriate m, U-function, and the DS(G, m) estimate, as shown in Figure 12.

E. SOFTWARE

We provide an `R` package, `BayesGOF` [17] to perform all the tasks outlined in the paper. We now summarize the main functions and their usage for the Rat binomial data example:

```
 #Phase I: Modeling
library("BayesGOF")
data(rat)
rat.start <- gMLE.bb(rat$y, rat$n)$estimate
rat.ds <- DS.prior(rat, g.par = rat.start, family = "Binomial")
plot(rat.ds, plot.type = "Ufunc") # Figure 1(a)
plot(rat.ds, plot.type = "DSg") # Figure 2(a)
```

The package also provide functionalities for Macro and MicroInference:

```
 #Phase II: Inference
rat.ds.macro <- DS.macro.inf(rat.ds, num.modes = 2, method = "mode")
plot(rat.ds.macro) # Figure 3(a)
rat.ds.pos <- DS.micro.inf(rat.ds, y.0 = 4, n.0 = 14)
plot(rat.ds.pos)  # Figure 5(b)
```

We hope this software will encourage applied data scientists to apply our method for their real problems.

F. DATA CATALOGUE

Table 6: List of datasets by distribution family and sources. They are sorted first by family, then according to k: from large to small-scale studies.

Dataset	# Studies (k)	Family	Sources
Surgical Node	844	Binomial	Efron (2016) [6]
Rolling Tacks	320	Binomial	Beckett and Diaconis (1994) [18]
Rat Tumor	70	Binomial	Gelman et al. (2013, Ch. 5) [14]
Terbinafine	41	Binomial	Young-Xu and Chan (2008) [19]
Naval Shipyard	5	Binomial	Martz et al. (1974) [20]
Galaxy	324	Gaussian	De Blok et al.(2001) [21]
Ulcer	40	Gaussian	Sacks et al.(1990) [22]
Arsenic	28	Gaussian	Willie and Berman (1995) [23]
Insurance	9461	Poisson	Efron and Hastie (2016) [24]
Child Illness	602	Poisson	Wang (2007) [10]
Butterfly	501	Poisson	Fisher et al. (1943) [25]
Norberg	72	Poisson	Norberg(1989) [26]

G. THE ROBBINS' PUZZLE

In Section 3.3 of the main paper, we presented a simulated scenario (Pharma-example) that demonstrated the power of the DS Elastic-Bayes estimate when there is significant prior-data conflict. Here we include further comparisons with two recent methods: Efron's Bayesian deconvolution (implemented in the `deconvolveR` package), and Koenker's NPMLE (implemented in the `REBayes` package).

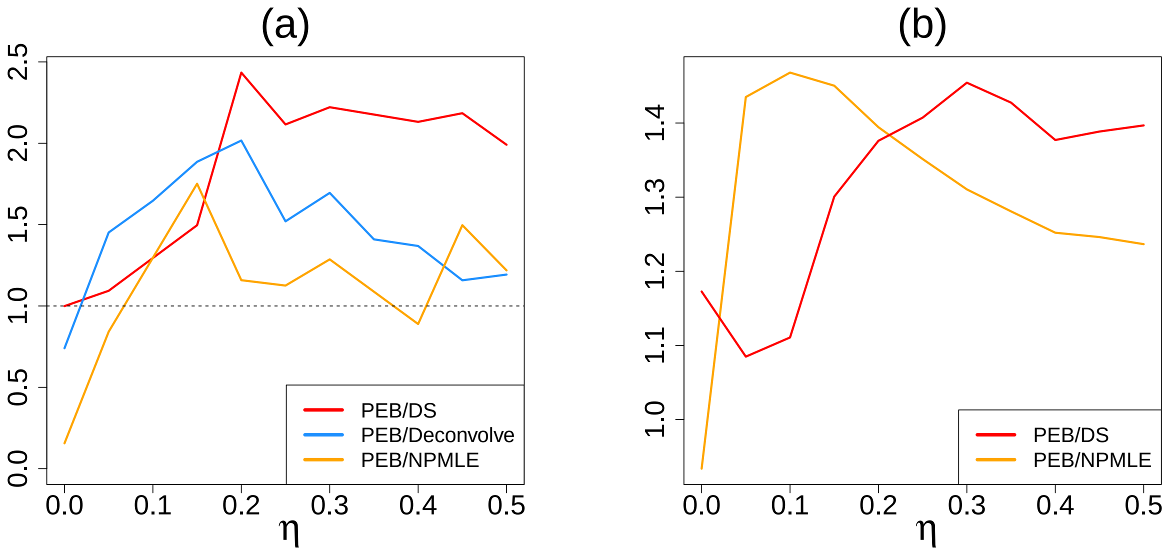

Figure 13: Results of two separate simulations comparing DS with other methods. In (a), the MSE ratios for PEB to empirical Bayes deconvolution (PEB/Dec; blue), PEB to Kiefer-Wolfowitz NPMLE using REBayes `Bmix` (PEB/NPMLE; orange) and PEB to DS (PEB/DS; red) with respect to η. Panel (b) shows the ratio of empirical risks after applying both DS and NPMLE methods to Robbins' 'compound decision' problem.

Example 1. Here we will operate under the exact settings presented in Section 3.3. Figure 13(a) shows that as η increases, DS tends to outperform the other two methods, although Deconvolve performs superbly for η smaller than 0.15. Two specially interesting extreme cases are $\eta = 0$ and $\eta = 0.5$. The first scenario describes the situation when the underlying parametric *beta* distribution is the right choice for the prior where, as expected, the Stein's parametric shrinkage estimator dominates other nonparametric approaches. On the other hand, the $\eta = 0.5$ is a complicated situation where $\pi(\theta) = \frac{1}{2}\text{Beta}(5, 45) + \frac{1}{2}\text{Beta}(30, 70)$, and consequently, the parametric EB [PEB] is less efficient compared to the nonparametric ones. The most interesting and surprising result, however, comes from DS Elastic-Bayes, which acts like the Stein prediction formula when underlying parametric assumption is correct (the null $\eta = 0$ case) but adapts itself non-parametrically in a completely automated manner when the true $\pi(\theta)$ deviates from the assumed g, thereby elegantly addressing the robustness-efficiency puzzle of Robbins [27].

Example 2. Next, we investigate the prediction problem originally introduced by Robbins [28] and discussed in Gu and Koenker [8]. We observe $Y_i = \theta_i + \epsilon_i$, $i = 1 \cdots k$, where $\epsilon_i \overset{\text{ind}}{\sim}$ Normal$(0, 1)$, and $\theta_i = \pm 1$ with probability η and $1 - \eta$ respectively. Our goal is to estimate the k-vector $\theta \in \{-1, 1\}^k$ under the loss $\mathrm{L}(\hat{\theta}, \theta) = k^{-1} \sum_{i=1}^{k} |\hat{\theta}_i - \theta_i|$. For comparison purpose, we computed the ratio of PEB empirical risk† to the the DS method (EB/DS) and to the NPMLE estimator (EB/KW) for $k = 1000$. Figure 13(b) shows a very interesting result: Kiefer-Wolfowitz NPMLE method performs significantly better than the DS-elastic Bayes when $0 < \eta < .2$. While for other values of η, including η equals to zero point, our micro-estimation procedure demonstrates tremendous promise. This further validates the flexibility and adaptability of our technique even in the discrete settings.

H. EXAMPLE WITH COVARIATES

The 'Bayes via goodness-of-fit' methodology can easily accommodate additional covariates. We demonstrate this capability using the following example.

The Norberg Example. The Norberg insurance dataset [26] consists of $k = 72$ Norwegian occupational categories, where y_i denotes the number of claims made against a policy. Additionally, we have the total number of years each group was exposed to risk E_i; when normalized by a factor of 344, E_i gives the expected number of claims during a contract period. Similar to Norberg [26], we assume $Y_i \sim \text{Poisson}(\theta_i E_i)$. Given the normalized E_i, we interpret θ_i as the occupational-specific rate of risk.

DS-Bayes analysis yields the following estimated prior, where g is the conjugate gamma prior with MLE $\alpha = 6.02$ and $\beta = 0.20$:

$$\widehat{\pi}(\theta) \;=\; g(\theta; \alpha, \beta)\big[1 - 0.70T_1(\theta; G) + 0.83T_2(\theta; G) - 0.53T_3(\theta; G)\big]. \tag{2}$$

In Figure 14(a), the U-function clearly indicates potential prior-data conflict when using $\pi(\theta) = \text{Gamma}(6.02, 0.20)$. Figure 14(b) displays the DS prior (red) along with the parametric EB (blue) and the Kiefer-Wolfowitz NPMLE estimate (green). We see a definite bimodality for $\widehat{\pi}(\theta)$, indicating that there are two distinct groups of risk profiles. The macroinference plot in Figure 14(c) reinforces the structured heterogeneity of the data. In terms of risk-profile, we consider the mode at 0.59 as occupational categories with comparatively lower risk; these are occupations less likely to make a claim based on their risk exposure. The mode at 1.46 represents those occupations at a higher risk, thus more likely

†Mean loss is computed over 500 replications.

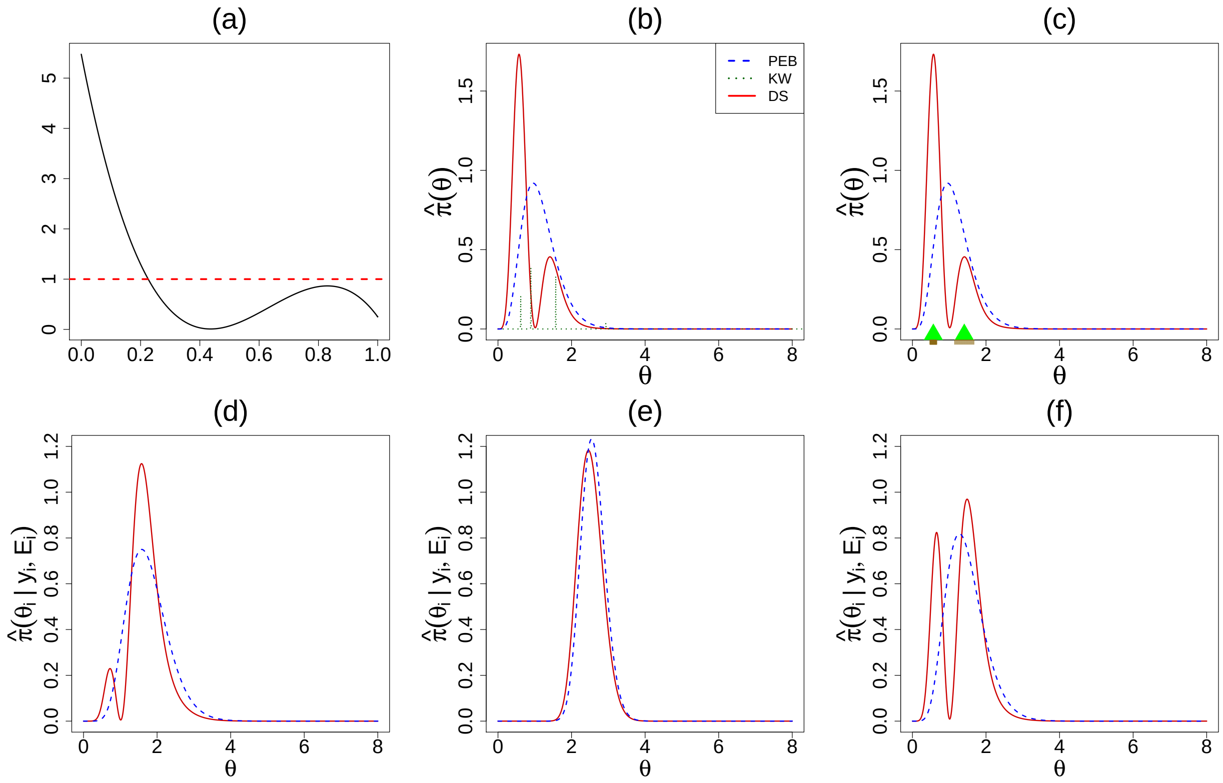

Figure 14: Demonstration of DS-Bayes with covariates on the Norberg insurance dataset. In (a), we display the U-function. Panel (b) shows the DS-prior (red), the PEB prior (blue) and the Kiefer-Wolfowitz NPMLE prior (green). Panel (c) shows the macroinference with standard errors (using smooth bootstrap): two modes located at $0.57(\pm 0.094)$ and $1.41(\pm 0.261)$. Panels (d) through (f) show microinference for occupational groups 13, 22, and 53 (respectively).

to make a claim based on their exposure. Of particular interest are panels (d), (e), and (f). These panels show the microinference for three specific occupational groups: group 13 ($Y_{13} = 4$, $E_{13} = 0.45$), group 22 ($Y_{22} = 57$, $E_{22} = 19.1$), and group 53 ($Y_{53} = 2$, $E_{53} = 0.25$). In Figure 14(d), we have an occupational category that identifies as higher risk with a small lower risk component. The unimodality in Figure 14(e) clearly indicates that category is a higher risk of claim based on exposure. Finally, the occupational category in Figure 14(f) is tricky. Here, we have bimodality with an almost equal probability of being a high or low-risk occupation. While the other two groups provide clear alternatives for an insurance company, the occupational group 53 needs the company's judgment in assigning the policy.

I. MAXIMUM-ENTROPY ENHANCEMENT

For more enhanced result, we offer an extension to maximum entropy DS(G, m) model, which assumes the following representation of the prior distribution:

$$\breve{\pi}(\theta) \;=\; g(\theta;\alpha,\beta)\,\exp\Big[c_0 + \sum_j c_j\,T_j(\theta;G)\Big], \tag{3}$$

where c_0 is some normalizing constant and the c_j's are the LP-maximum entropy coefficients. The following algorithm outlines the process to solve for the unknown c_j's starting from the $\mathcal{L}^2$ estimate.

Orthogonal Series to Maximum Entropy Estimator

`Step 0.` Input: BIC-smoothed LP-Fourier ($\mathcal{L}^2$) coefficients $\widehat{\mathrm{LP}}[j;G,\Pi]$, $j = 1,\ldots,m$.

`Step 1.` Define the set $\mathcal{J} = \Big\{j : |\widehat{\mathrm{LP}}[j;G,\Pi]| > 0\Big\}$, collection of j's for which we have significant non-zero $\mathcal{L}^2$ orthogonal coefficients.

`Step 2.` To estimate the maximum entropy coefficients c_j in $\breve{\pi}(\theta)$ of (3), solve the following sets of moment equality constraints:

$$\widehat{\mathrm{LP}}[j;G,\Pi] = \int T_j(\theta;G)\breve{\pi}(\theta)d\theta, \quad \text{for } j \in \mathcal{J}. \tag{4}$$

`Step 3.` Output: $\big(\hat{c}_0, \{\hat{c}_j\}_{j\in\mathcal{J}}\big)$; accordingly the estimated maximum entropy $\breve{d}$ and $\breve{\pi}$.

Two Data Examples. Here we carry out the maximum entropy analysis for `rat` (binomial variate) and `galaxy` data (normal variate). The `galaxy` data consists of $k = 324$ observed rotation velocities y_i and their uncertainties of Low Surface Brightness (LSB) galaxies [21].

(a) Rat Tumor data, g is beta distribution with MLE $\alpha = 2.30$, $\beta = 14.08$:

$$\breve{\pi}(\theta) = g(\theta;\alpha,\beta)\exp\big[-0.13 - 0.52T_3(\theta;G)\big]. \tag{5}$$

(b) Galaxy data, g is normal distribution with MLE $\mu = 85.5$, $\tau^2 = 3304$:

$$\breve{\pi}(\theta) = g(\theta;\mu,\tau^2)\exp\big[-0.15 + 0.26T_3(\theta;G) - 0.28T_4(\theta;G) + 0.46T_5(\theta;G)\big]. \tag{6}$$

The resulting LP-maximum-entropy DS(G,m) priors are shown in Figure 15. In both examples, we see the maximum entropy estimates (green dashed lines) are very similar to the $\mathcal{L}^2$ with some adjustments to the modal shapes.

The `BayesGOF` package in R implements this algorithm as an option for the `DS.prior` function. The following code demonstrates how to generate both the $\mathcal{L}^2$ and maximum entropy representations of the LP coefficients for both the rat tumor and galaxy data sets.

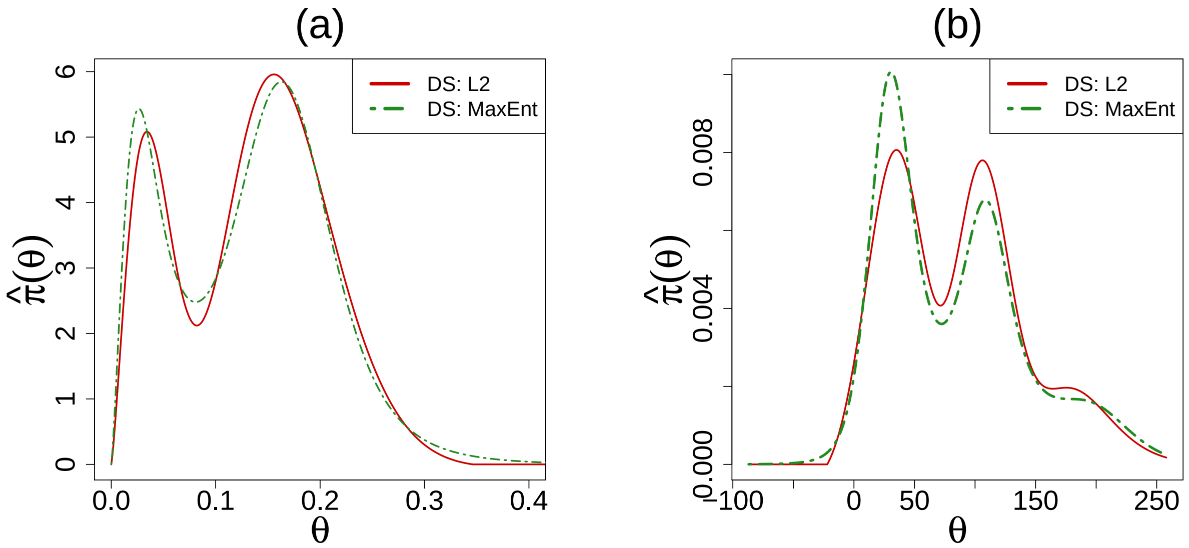

Figure 15: Comparison of $\mathcal{L}^2$ (solid red line) and maximum entropy (two-dash green line) estimates of DS prior. Panel (a) shows the comparison for the `rat` tumor data, while panel (b) illustrates the difference (in modal shapes) for the `galaxy` data.

```
library(BayesGOF)
#---Rat Tumor Data
data(rat)
rat.start <- gMLE.bb(rat$y, rat$n)$estimate
rat.ds.L2 <- DS.prior(rat, max.m = 4, g.par = rat.start,
                      family = "Binomial", LP.type = "L2")
## Shown in Figure 15(a) as solid red line
rat.ds.ME <- DS.prior(rat, max.m = 4, g.par = rat.start,
                      family = "Binomial", LP.type = "MaxEnt")
## Shown in Figure 15(a) as two-dashed green line
#---Galaxy Data
data(galaxy)
gal.start <- gMLE.nn(galaxy$y, galaxy$se)$estimate
gal.ds.L2 <- DS.prior(galaxy, max.m = 5, g.par = gal.start,
                      family = "Normal", LP.type = "L2")
## Shown in Figure 15(b) as solid red line
gal.ds.ME <- DS.prior(galaxy, max.m = 5, g.par = gal.start,
                      family = "Normal", LP.type = "MaxEnt")
## Shown in Figure 15(b) as two-dashed green line
```

References

[1] Berger, J. O. An overview of robust Bayesian analysis (with discussion). *Test* **3**, 5–124 (1994). DOI 10.1007/BF02562676.

[2] Berger, J. & Berliner, L. M. Robust Bayes and empirical Bayes analysis with ε-contaminated priors. *The Annals Stat.* 461–486 (1986).

[3] Morris, C. N. Parametric empirical Bayes inference: theory and applications. *J. Am. Stat. Assoc.* **78**, 47–55 (1983).

[4] Efron, B. Robbins, empirical Bayes and microarrays. *The Annals Stat.* **31**, 366–378 (2003).

[5] Efron, B. Empirical Bayes methods for combining likelihoods. *J. Am. Stat. Assoc.* **91**, 538–550 (1996).

[6] Efron, B. Empirical Bayes deconvolution estimates. *Biom.* **103**, 1–20 (2016).

[7] Efron, B. Why isn't everyone a Bayesian? *The Am. Stat.* **40**, 1–5 (1986).

[8] Gu, J. & Koenker, R. On a problem of Robbins. *Int. Stat. Rev.* **84**, 224–244 (2016).

[9] Laird, N. Nonparametric maximum likelihood estimation of a mixing distribution. *J. Am. Stat. Assoc.* **73**, 805–811 (1978).

[10] Wang, Y. On fast computation of the non-parametric maximum likelihood estimate of a mixing distribution. *J. Royal Stat. Soc. Ser. B (Statistical Methodol.* **69**, 185–198 (2007).

[11] Ferguson, T. S. A Bayesian analysis of some nonparametric problems. *The Annals Stat.* 209–230 (1973).

[12] Liu, J. S. Nonparametric hierarchical Bayes via sequential imputations. *The Annals Stat.* 911–930 (1996).

[13] Gelman, A., Jakulin, A., Pittau, M. G. & Su, Y.-S. A weakly informative default prior distribution for logistic and other regression models. *The Annals Appl. Stat.* 1360–1383 (2008).

[14] Gelman, A. *et al. Bayesian Data Analysis, Third Edition.* Chapman & Hall/CRC Texts in Statistical Science (Taylor & Francis, 2013).

[15] Berger, J. O. Bayesian analysis: A look at today and thoughts of tomorrow. *J. Am. Stat. Assoc.* **95**, 1269–1276 (2000).

[16] R Core Team. *R: A Language and Environment for Statistical Computing.* R Foundation for Statistical Computing, Vienna, Austria (2017).

[17] Mukhopadhyay, S. & Fletcher, D. *BayesGOF: Bayesian Modeling via Goodness of Fit* (2018). R package version 3.1.

[18] Beckett, L. & Diaconis, P. Spectral analysis for discrete longitudinal data. *Adv. Math.* **103**, 107–128 (1994).

[19] Young-Xu, Y. & Chan, K. A. Pooling overdispersed binomial data to estimate event rate. *BMC Med. Res. Methodol.* **8**, 58 (2008).

[20] Martz, H. & Lian, M. Empirical Bayes estimation of the binomial parameter. *Biom.* **61**, 517–523 (1974).

[21] De Blok, W., McGaugh, S. S. & Rubin, V. C. High-resolution rotation curves of low surface brightness galaxies II. Mass models. *The Astron. J.* **122**, 2396 (2001).

[22] Sacks, H. S., Chalmers, T. C., Blum, A. L., Berrier, J. & Pagano, D. Endoscopic hemostasis: an effective therapy for bleeding peptic ulcers. *J. Am. Med. Assoc.* **264**, 494–499 (1990).

[23] Willie, S. & Berman, S. Ninth round intercomparison for trace metals in marine sediments and biological tissues. *NRC/NOAA* (1995).

[24] Efron, B. & Hastie, T. *Computer Age Statistical Inference*, vol. 5 (Cambridge University Press, 2016).

[25] Fisher, R. A., Corbet, A. S. & Williams, C. B. The relation between the number of species and the number of individuals in a random sample of an animal population. *The J. Animal Ecol.* 42–58 (1943).

[26] Norberg, R. Experience rating in group life insurance. *Scand. Actuar. J.* **1989**, 194–224 (1989).

[27] Robbins, H. An empirical Bayes estimation problem. *Proc. Natl. Acad. Sci.* **77**, 6988–6989 (1980).

[28] Robbins, H. Asymptotically subminimax solutions of compound statistical decision problems. In *Proceedings of the Second Berkley Symposium on Mathematical Statistics and Probability*, vol. I, 131–149 (Berkeley: University of California Press, 1951).
